# The versatile emodin: A natural easily acquired anthraquinone possesses promising anticancer properties against a variety of cancers

**DOI:** 10.7150/ijbs.70447

**Published:** 2022-05-16

**Authors:** Qing Zhang, Wen Wen Chen, Xue Sun, Die Qian, Dan Dan Tang, Li Lin Zhang, Mei Yan Li, Lin Yu Wang, Chun-Jie Wu, Wei Peng

**Affiliations:** 1 State Key Laboratory of Southwestern Chinese Medicine Resources, School of Pharmacy, Chengdu University of Traditional Chinese Medicine, Chengdu 611130, P.R. China.; 2 Department of Pharmacy, Chengdu Women's and Children's Central Hospital, School of Medicine, University of Electronic Science and Technology of China, Chengdu, 610091, P.R. China.

**Keywords:** Emodin, Apoptosis, Metastasis and invasion, Cycle arrest, Drug resistant, Autophagy

## Abstract

Cancers are generally recognized as the leading cause of death and a predominant barrier to prolonging life expectancy in both developed and developing countries. Emodin is a typical anthraquinone derivative from various plants that exhibits a wide spectrum of biological activities, such as anticancer, antibacterial, hepatoprotective and anti-inflammatory activities. Much previous preclinical evidence has demonstrated that emodin exhibits reliable effects on several cancer types, including lung cancer, liver cancer, colon cancer, breast cancer, pancreatic cancer, leukemia, cervical cancer, and ovarian cancer, *etc*. The related molecular mechanisms corresponding to the anticancer activities of emodin are involved in the induction of apoptosis, inhibition of cell proliferation, enhanced reactive oxygen species (ROS) accumulation, and induction of autophagy, *etc*. In the present review, we summarized the sources, anticancer properties *in vitro* and *in vivo*, molecular mechanisms, metabolic transformation and toxicities of emodin. In addition, we also discussed the limitations of the present investigations of emodin against cancers and gave some perspectives for them, which would be beneficial for the further exploration and development of this natural compound as a clinical cancer drug.

## Introduction

Currently, epidemiological evidence reveals that the incidence and mortality of cancers are rapidly rising worldwide, and cancers are also generally recognized as the leading causes of death and a predominant barrier to prolonging life expectancy in both developed and developing countries 1, 2. Importantly, it is estimated that there were more than 180,000,000 new cancer cases and 100,000,000 cancer-caused deaths each year nowadays 1,3. Furthermore, it has been reported that regardless of sex, lung cancer is the most common cancer clinically and the primary cause of cancer-related death, followed by breast cancer, colorectal cancer, prostate cancer, stomach cancer, liver cancer, esophageal cancer, cervical cancer, thyroid cancer and bladder cancer, and these ten cancer types account for approximately 65% of newly occurring cancer cases 1, 4. In recent years, although the diagnosis and treatment of cancers have improved greatly, the 5-year survival rate of cancer patients is still relatively poor due to the gradually appearing drug resistance of cancer cells and the excessively high price of anticancer drugs, particularly for developing areas 5,6. In addition to surgery and radiotherapy, chemotherapy remains the predominant treatment for cancer, and the currently available anticancer drugs usually target fast-dividing cells, which could damage both the cancer cells and some normal cells, such as epithelial cells of the digestive tract, marrow cells and hair follicles, leading to serious side effects, including hair loss, myelosuppression, vomiting and diarrhea 5, 7. Therefore, finding more reliable and economical treatment strategies with low toxicity against cancers is urgently needed.

**Figure 1:**
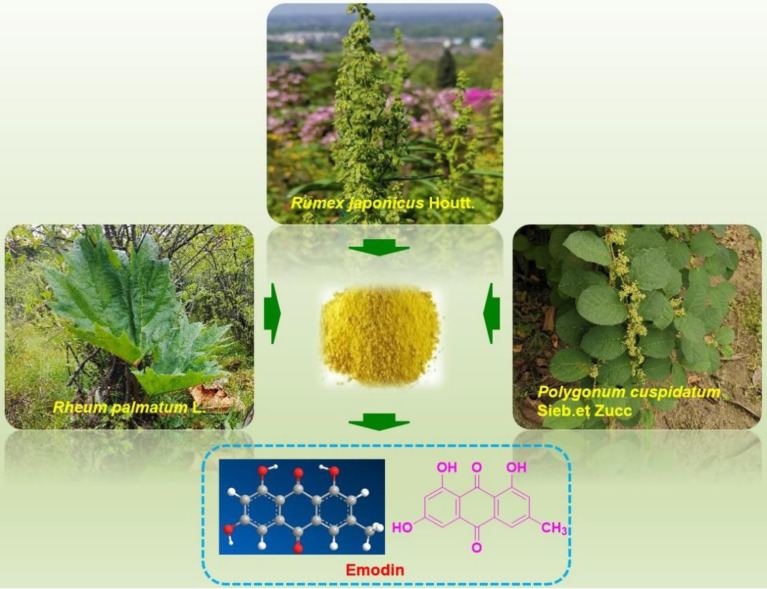
*Polygonum cuspidatum* and chemical structure of emodin.

Currently, an increasing number of studies have focused on the possible capacities of natural agents extracted from herbal medicines to prevent or cure cancers and have discovered many anticancer drugs, such as taxol, vincaleukoblastinum, and camptothecin, *etc.* 5, 8, 9. Emodin (chemical structure shown in Figure 1) is a typical anthraquinone derivative from herbal medicine that exhibits a wide spectrum of biological activities, such as anticancer, antibacterial, hepatoprotective and anti-inflammatory activities, according to previous investigations 10, 11. Much previous preclinical evidence has demonstrated that emodin exhibits reliable effects on several cancer types, including lung cancer, liver cancer, colon cancer, breast cancer, pancreatic cancer, leukemia, cervical cancer, and ovarian cancer 12, 13. The related molecular mechanisms corresponding to the anticancer activities of emodin are involved in the induction of apoptosis, inhibition of cell proliferation, enhanced reactive oxygen species (ROS) accumulation, and induction of autophagy 12, 14-16. In our previous works, we found that emodin exists in *Polygonum cuspidatum* at a high amount and has interesting effects on anti-methicillin-resistant *Staphylococcus aureus* (MRSA) 17, 18. Furthermore, we have reported many natural extract/monomer anticancer agents with potential antitumor properties 7, 19-22; as part of our continuing investigations, we also found that emodin has significant anticancer activities against various cancer cell lines and might be a promising compound for development as an effective and economical anticancer drug with low toxicity.

Consequently, in the present review, we summarized the sources, anticancer properties including dose ranges tested* in vitro* and* in vivo*, metabolic transformation and toxicities of emodin, which would be beneficial for the further exploration and development of this natural compound as a clinical cancer drug (Table 1).

## Resources of emodin

Emodin is a time-honored natural compound that was first reported from plants of the *Rheum* genus by *von Warren de la Rue* and *Hugo Nuller* in the year of 1857 23. Emodin, 3-methyl-1,6,8-trihydroxyanthraquinone, was reported to be the most ubiquitous natural anthraquinone in the natural world and can be found in many higher plants, lichens and even fungi 10, 11, 13, 24, 25. Emodin commonly exists abundantly in plants of the *Polygonaceae* family, such as *Polygonum multiflorum*, *Rheum palmatum*, *Rheum tanguticum*, *Rheum officinale*, *Polygonum Cuspidatum*, *Rumex japonicus*, *Rumex hydrolapathum*, *Rumex scutatus*, *Rumex confertus, Rumex altissimus*, *Rumex crispus*, *Rumex stenophyllus*, *Rumex arifolius*, *Rumex patientia*, *Rumex sanguineus*, *Rumex brownii*, *Rumex pulcher*, *Rumex acetosa*, *Rumex conglomeratus*, *Rumex acetosella*, *Rumex nepalensis*, *Rumex maritimus*, *Rumex alpinus*, *Rumex palustris*, and *Rumex obtusifolius* 11, 24, 26, 27. Additionally, emodin was also reported in plants in several other families, such as *Aloe vera* (*Asphodelaceae*), *Acorus tatarinowii* (*Araceae*), *Cassia obtusifolia* (*Leguminosae*), *Cassia occidentalis* (*Leguminosae*), *Eriocaulon buergerianum* (*Eriocaulaceae*), *Dendrobium thyrsiflorum* (*Orchidaceae*), *Fibraurea tinctoria* (*Aristolochiaceae*), *Coptis chinensis* (*Ranunculacea*), *Scutellaria baicalensis* (*Lamiaceae*), and *Isatis indigotica* (*Brassicaceae*) 10,13. Among these herbs and plants,* Polygonum cuspidatum* might possess the highest content of emodin, with contents ranging from 0.6% to 2.3%, and it is currently the primary resource for the isolation of emodin 28. The extraction of emodin from plants is inefficient and requires a large amount of plant material. Fortunately, the yield of emodin can be greatly increased through synthetic methods. Through biosynthetic pathway, emodin was synthesized by acetyl CoA carboxylase (ACC1), polyketo synthase (PKS), thiesterase (MβL-TE) decarboxylase (DC) and other enzymes, starting from Pyruvate 29. In addition, some efficient chemical synthesis methods also enrich the sources of emodin.

## Chemical properties of emodin

The molecular formula of emodin is C_15_H_10_O_5_, and the molecular weight is 270.23. Emodin is commonly orange powder, and the melting point range is 256-257 °C. Emodin contains multiple hydroxyl and carbonyl groups in its structure, which can chelate with metal ions in biological target enzymes to form relatively stable chelates, which is also one of the important reasons for its wide biological activity. Emodin can be used as a reversible binding agent for DNA. Its aromatic ring plane structure can be embedded and superimposed between the double helix base pairs of DNA, combine with DNA, destroy the normal helix structure of DNA, and interfere with the role of DNA binding enzymes such as DNA topoisomerase and DNA polymerase. Simply put, emodin can capture the DNA-topoisomerase II lysable complex and form the emodin-DNA-topoisomerase terpolymer complex to stabilize it and interfere with the reconnection reaction of DNA breakpoints. The interference of emodin with DNA polymerase may not only inhibit the elongation of the DNA chain from 5' to 3' but also prevent DNA error correction by inhibiting the shearing process of mismatched residues (hydrolysis of phosphate diester bond). In conclusion, emodin can inhibit DNA replication and exert pharmacological activity through the chimerism of DNA. At the same time, emodin's DNA-damaging activity also involves electron transfer chemistry, which is consistent with other anthracycline antibiotics. Under the action of cytochrome CYP450 reductase, emodin undergoes one-electron reduction to produce superoxide (O_2_^-^), which in turn produces a large number of hydroxyl radicals, causing DNA chain breaks 30. It is well known that the typical anthraquinone tricyclic aromatic structure of emodin results in poor water solubility (0.07 mg/mL) and low bioavailability, which limits its clinical application 31. However, by chemically modifying the structure of emodin and introducing hydrophilic groups such as -OH and -NH-, it is possible to improve its water solubility and bioavailability. In 2004, Teich et al. reported on the synthesis and activity of emodin derivatives and found that emodin derivatives with different amine substitutions at four sites exhibited stronger antitumor fine activity than emodin itself 32. Shao *et al*. synthesized a series of quaternary ammonium derivatives of emodin by introducing quaternary ammonium salts at sites 6 and 3 of emodin, tested the biological activities of these compounds, and found that the derivatives had strong anti-proliferation ability against the HepG2 GC-823 AGS cell line but low toxicity against the HELF normal cell line 33. A recent study reported that the new semisynthetic anthraquinone derivatives with the NαFmoc-l-Lys and ethynyl group NαFmoc-l-Lys synthesized from emodin significantly increased the inhibition rate of HT-29 and HeLa cells 34. In addition, dosage form modification of emodin by pharmaceutical means can also improve the bioavailability and pharmacological activity of emodin. Di et al. used piperine as a bioenhancer to inhibit the glucuronidation of emodin in the liver and intestine to improve its bioavailability 35. Emodin was modified by arginine-glycine-aspartic acid (RGD) to produce a targeted liposome that can effectively inhibit vasculogenic mimicry (VM) channel formation and metastasis in breast cancer tumors and increase the antitumor effect of emodin 36. A recent study found that a transfer form of nano-emodin, a novel sonar-responsive nanomaterial, was synthesized to enhance the accumulation and penetration of nanoparticles and could be used as an effective intervention for the treatment of head and neck squamous cell carcinoma (HNSCC) 37. In addition, Krajnovi´c *et al*. used mesoporous silica as a carrier to transport emodin, which can ensure the release of emodin in the extremely acidic environment of the stomach, prevent the photodecomposition of emodin, and improve the anti-proliferation and pro-apoptotic effects of emodin on a variety of tumor cells 38.

## Anticancer effects of emodin

### Lung cancer

**Figure 2:**
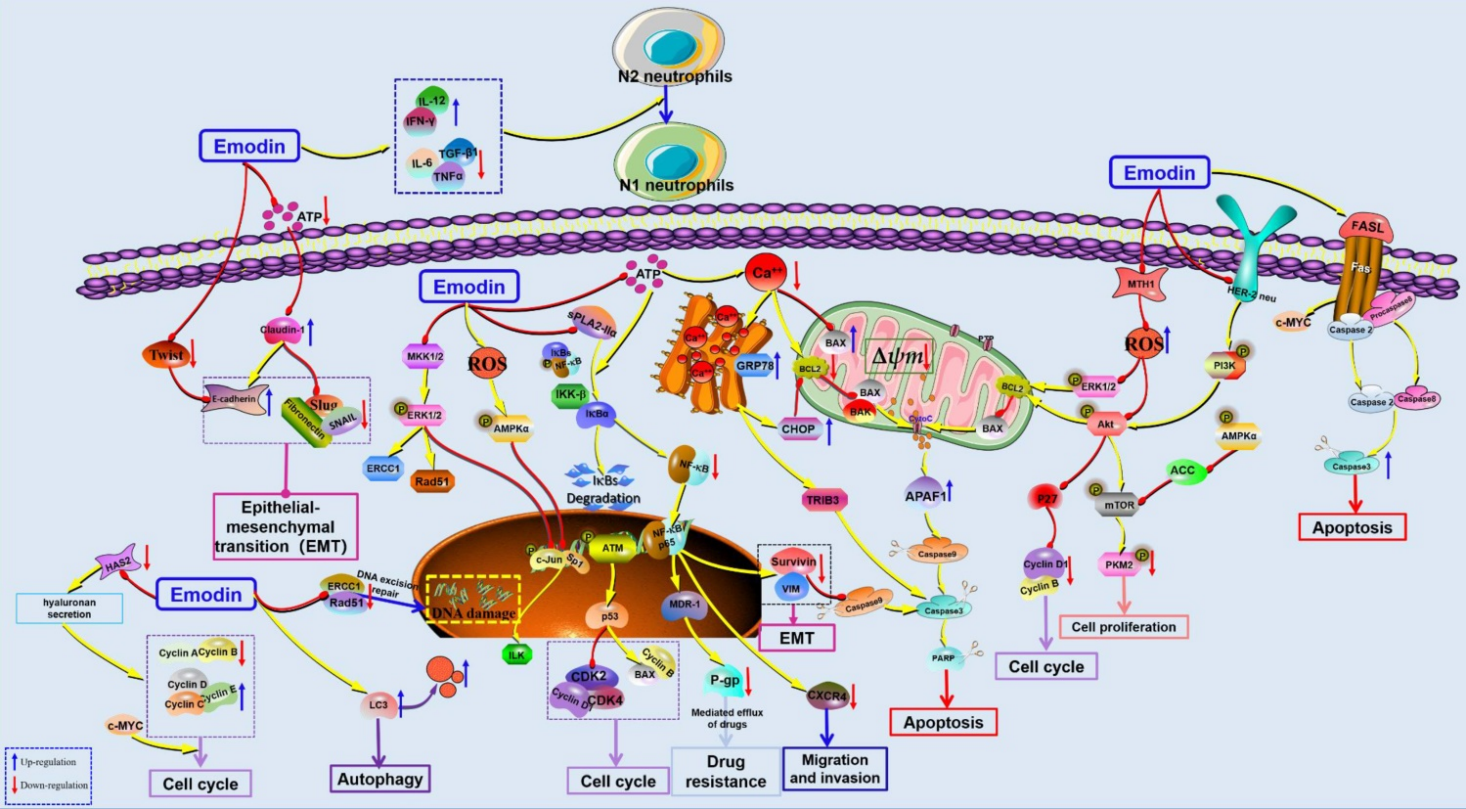
The potential mechanisms for antitumor effect of emodin against lung cancer.

Lung cancer currently ranks as the most diagnosed malignant cancer type clinically and remains the leading cause of cancer-related death 1,2. Currently, increasing scientific evidence has demonstrated that emodin shows potential anticancer effects against lung cancers *in vivo* and *in vitro via* inhibition of cell proliferation and metastasis, induction of apoptosis and cell cycle arrest, and increase in ROS, *etc.* (Figure 2). In 1996, a study by Zhang and Hung indicated that emodin possesses significant anti-proliferative activity and could also reverse the drug resistance of HER-2/neu-overexpressing lung cancer cells to chemotherapeutic drugs (cisplatin, doxorubicin or VP16) *via* inhibition of the protein tyrosine kinase 39. Later, in 2010, Ko *et al*. reported that emodin exhibited strong cytotoxicity against lung cancer cell lines via suppression of excision repair cross-complementing gene (ERCC) 1 and Rad 51 40; similarly, He *et al.* reported that emodin exerted suppressive activity against the proliferation of A549 cells in a concentration-dependent manner, and the possible molecular mechanisms involved the downregulation of ERCC1 and Rad51 41. Another report by Ko *et al*. suggested that emodin could also strengthen cisplatin-induced cytotoxicity in lung cancer cells *via* ERCC1 downregulation and ERK1/2 inactivation 42. In addition, Tang *et al*. revealed that the possible mechanisms regarding the anticancer effects of emodin against lung cancer might involve the inhibition of ILK expression *via* an increase in the phosphorylation of AMPKα and ERK1/2 and the suppression of Sp1 and c-Jun 43. Recently, a research report by the same research team of Tang *et al*. demonstrated that emodin could not only inhibit cell growth but also induce cell cycle arrest at G2/M phase in A549 cells, and the potential mechanism is associated with activation of PPARγ and the AMPKα/MEK/ERK signaling pathway, downregulation of Sp1 and upregulation of IGFBP1 44. Li et al. found that emodin can induce G1/G0 phase arrest of lung cancer cells by regulating the secretion of hyaluronic acid 45.

Furthermore, the induction of apoptosis was also observed as an important reason corresponding to its anticancer effects against lung cancer. In 2001, Lee reported that emodin-induced cell death is closely associated with mitochondria-dependent apoptosis in CH27 cells 46. Later, Li *et al*. reported that emodin induced A549 cell growth inhibition and apoptosis through extrinsic apoptotic pathways and induction of cell cycle arrest 47. Another research study showed that emodin could reduce the viability of A549 cells in a concentration-dependent manner through the induction of apoptosis *via* the activation of ER stress and the TRIB3/NF-κB pathway, and the antitumor effect of emodin was confirmed in an A549 tumor-bearing BALB/c nude mouse model *in vivo* 48. It has been reported that emodin can also induce cancer cell apoptosis by enhancing intracellular ROS levels. Lai *et al*. revealed that emodin could induce mitochondria-dependent apoptosis in A549 cells by activating the ROS-elicited ATM-p53-Bax signaling pathway 15. Similar to the works of Lai *et al*., the findings of another study by Su *et al*. suggested that emodin-induced apoptosis in A549 cells is closely correlated with emodin-mediated ROS generation and reduced ∆*Ψ*_m_ 49. Previous studies have also found that emodin can significantly induce apoptosis of A549 cells 50. The 18 kD human MutT homolog 1 (MTH1) protein, also known as Nudix hydrolase 1 (NUDT1), is a major intracellular pyrophosphatase that prevents oxidative nucleotide precursors from misfitting into genomic DNA, preventing damage and cell death 51. A recent study found that emodin can act as an MTH1 inhibitor to induce ROS production and promote DNA damage and apoptosis of tumor cells 52. Zhang *et al*. also found that emodin could significantly inhibit the proliferation of NSCLC *in vitro* and *in vivo* but had low cytotoxicity to normal lung cell lines. The mechanism was related to the inhibition of mTOR and AKT and the activation of the AMPK pathway 53.

In 2012, He *et al*. reported that after treatment with emodin, some typical autophagosomes could be observed in A549 cells 41; recently, Haque *et al*. indicated that emodin-induced autophagy has a close relationship with mutation-independent p53 aggregation 16. Metastasis is undoubtedly one of the worst outcomes of cancer development. In 2012, emodin suppressed CXCL12-induced A549 cell migration and invasion, and the molecular mechanism involved in the downregulation of CXCR4 and HER2 expression 54. In addition, Wang *et al*. demonstrated that emodin suppressed ATP-induced proliferation and migration by suppressing the P2Y receptor and Ca^2+^-dependent NF-κB pathway 55. Furthermore, Ying *et al*. suggested that emodin can inhibit epithelial-mesenchymal transition (EMT), cell proliferation, migration and invasion of lung cancer cells, as well as effectively reverse the resistance of H69AR to Dox. The possible mechanism is involved in suppressing the expression of Twist, Snail & Slug and inhibiting the activation of NF-κB 56. In addition, Peng *et al*. found that emodin can be used as a chemotherapy sensitization agent to enhance the antitumor effect of cisplatin 57. Similarly, emodin can also increase the antitumor effect of paclitaxel *in vivo* and *in vitro* 58. Teng et al. also found that emodin can reverse cisplatin resistance in A549 cells by regulating the NF-κB pathway 59. Li *et al*. found that emodin can selectively inhibit N2 neutrophils to prevent hypercoagulation and lung carcinogenesis 60. Recently, Zhang *et al*. reported that emodin could induce apoptosis in NSCLC cells, and the related molecular mechanisms are correlated with the downregulation of the sPLA2-Iia and NF-κB pathways 61.

### Hepatocellular carcinoma

**Figure 3:**
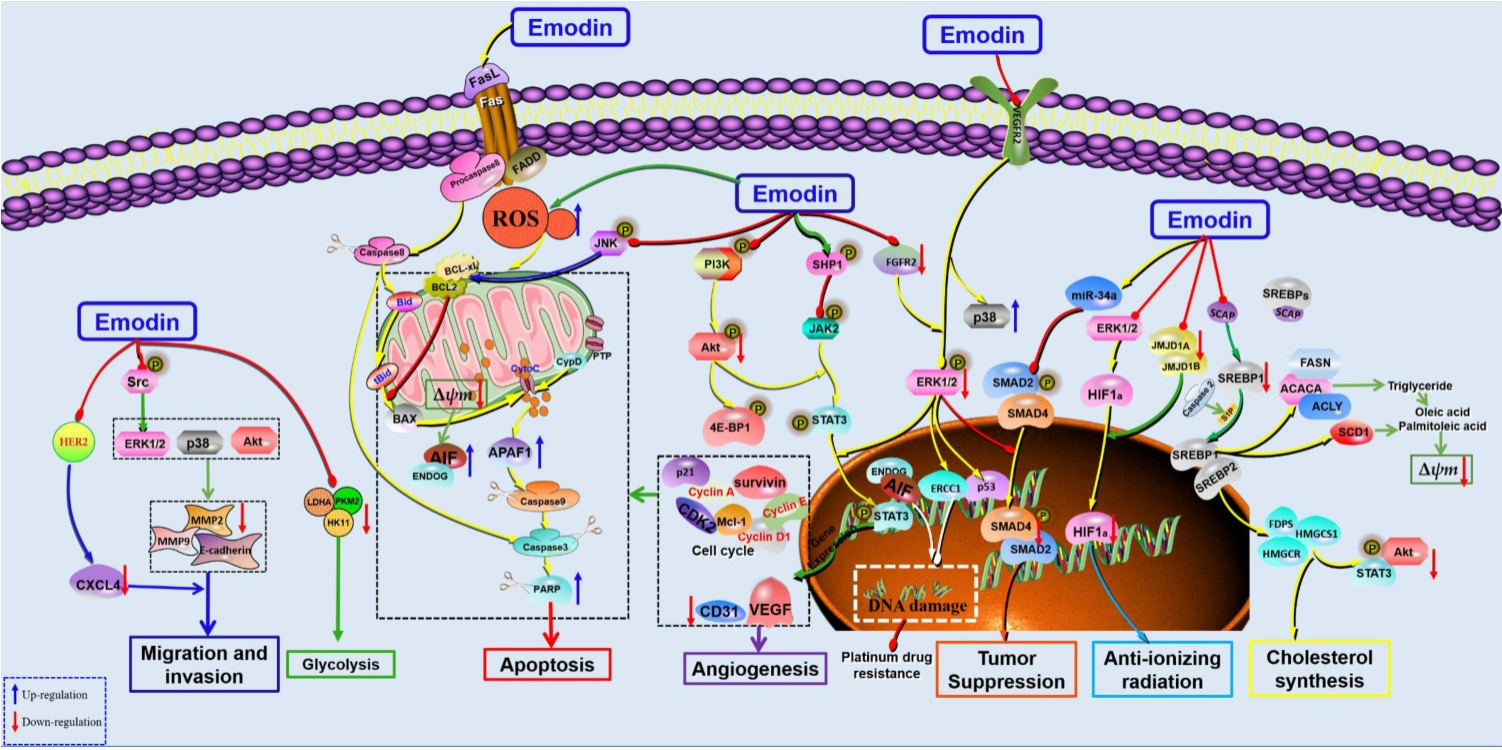
The potential mechanisms for antitumor effect of emodin against HCC.

Hepatocellular carcinoma (HCC), one of the world's leading malignancies, accounts for an estimated 800,000 deaths according to a 2017 study 62. Currently, the common treatment methods for liver cancer include surgical resection and radiofrequency ablation 63. Unfortunately, the high recurrence rate and metastatic tendency of liver cancer make these treatments less effective. Although chemical inhibitors or biologic agents are available for clinical use, they are often limited by tolerance and adverse reactions in patients. Therefore, there is an urgent need for safe and effective new drugs to prevent and treat liver cancer 64. HCC is another type of malignant cancer that has been comprehensively investigated with emodin, and previous studies have suggested that emodin possesses remarkable anticancer properties against various subtypes of liver cancers through the induction of apoptosis and cell cycle arrest, inhibition of cancer cell metastasis, and enhancement of DNA damage (Figure 3). Hsu et al. found that emodin induced G2/M phase arrest in HCC cells by upregulating CYP1A1, CYP1B1, GDF15, SERPINE1, SOS1, RASD1, MRAS, Cyclin A, cyclin B, Chk2, Cdk2 and P27 while downregulating NR1H4, PALMD, TXNIP, Cdc25c, and P21 65. Yu *et al*. also revealed that emodin can induce intracellular ROS accumulation in HCC cells, leading to increased cytochrome C in the cytoplasm and finally resulting in the activation of caspase-8 and caspase-9. In addition, emodin increased p53 protein levels and decreased NF-kB/P65 in HepG2 cells, and emodin directly binds to the BH3 domain of Bcl-2 by forming a hydrogen bond with Ala146 residues 66. In addition, Xing *et al*. found the cytotoxic effects of emodin against HepG2 cells are related to many endogenous metabolites, such as oxidative stress and amino acid and energy metabolism disorders 67.

In 2002, Jing *et al*. selected 3 hepatoma cell lines, human hepatocellular carcinoma cell lines, Mahlavu, PLC/PRF/5 and HepG2, to investigate the anticancer effects of emodin. The results showed that emodin could effectively induce apoptosis in human hepatoma cell lines. Further studies have found that emodin promotes apoptosis by inducing the production of ROS, which leads to a decrease in mitochondrial transmembrane potential (MMOP) and then activates caspase-9 and caspase-3 to achieve DNA fragmentation. In this study, oxidative stress was also found to play a key role in the proapoptotic effect of emodin 68. Interestingly, similar results were found in the studies of Xion *et al*. and Dong *et al*. 69, 70. Zhang *et al*. also reported that emodin induced mitochondrial dysfunction and apoptosis in HepG2 cells by regulating the mitochondrial matrix protein cyclophilin D (CyPD), providing a new perspective for emodin against liver cancer 71. Signal transducer and activator of transcription (STAT) is a cytoplasmic transcription factor highly expressed in various cancers 72, 73. Studies have found that inhibition of STAT3 can inhibit the proliferation of HCC cells and induce apoptosis 74, 75, suggesting that STAT3 might be a potential target for HCC. Subsequently, emodin showed significant antitumor effects against several HCC cell lines (HepG2, PLC/PRF/5, Hep3B and C3A cells) by inhibiting the activation and nuclear transcription of STAT3. Furthermore, another interesting paper revealed that emodin could inhibit the growth of *in situ* tumors in the liver and induce apoptosis by suppressing the activation of STAT3. In addition, this study first found that emodin can upregulate SHP-1 in HCC cells to inhibit the JAK/STAT signaling pathway 76. In 2016, emodin was reported to induce apoptosis of HCC cells through death receptor-mediated apoptosis and mitochondrial-dependent apoptosis by inhibiting the PI3K/Akt and MAPK signaling pathways 77. Interestingly, in addition to its antitumor effect against liver cancer, emodin can also improve liver and kidney functions in tumor-bearing mice, suggesting that emodin may improve quality of life in mice implanted with tumors 78. In 2019, emodin was shown to inhibit HCC by inhibiting VEGFR_2_-Akt-ERK_1/2_ signaling and upregulating miR-34A 79. Recently, Zhu et al. reported that the anticancer effect of emodin might be related to inhibiting graft tumor lipid metabolism via downregulation of SREBP cleavage-activating protein (SCAP) 80. Another study also found that emodin could inhibit the triglyceride level and fatty acid desaturation of HCC cells and induce apoptosis of HCC cells via regulation of SREBP1 81.

Migration is one of the main reasons leading to poor prognosis of tumor treatment, and chemokine receptors play important roles in tumor migration 82, 83. Manu *et al*. studied the effects of emodin on the chemokine CXCR4 in HCC cells and found that emodin can suppress CXCL12-induced migration in HCC cells by downregulating CXCR4 84. Using a HCC cell line with high malignant invasion potential, MHCC-97H, Lin *et al*. found that emodin can inhibit the migration and invasion of HCC cells by inhibiting the expression of matrix metalloproteinases (MMPs)-2 and -9 and regulating the MAPK and PI3K/Akt signaling pathways 85.

The anticancer effects of chemotherapy drugs are related to DNA damage in tumor cells, which is closely related to ERCC1 86, 87. Currently, the fibroblast growth factor receptor (FGFR) 2, a gene upstream of ERCC1, has also attracted much attention in anticancer studies 88. Chen et al. found that emodin can act as a tyrosine kinase inhibitor to promote DNA damage in HepG2 cells. Furthermore, emodin could also downregulate the expression of ERCC1 and p-ERK in HepG2 cells, which could be blocked by knockdown of FGFR2, suggesting that FGFR2 might be a potential target for the antitumor effects of emodin 89. In addition, emodin has been used as a radiosensitization agent in the treatment of HCC. It has been reported that the combination of emodin and radiotherapy can regulate the cycle progression of HCC cells and induce apoptosis, and the mechanism is related to upregulation of cleaved-PARP1 and downregulation of JMJD1A and JMJD2B 90. In addition, emodin can also enhance the anticancer effect of sorafenib on liver cancer cells 91. Similarly, Wang *et al*. reported that emodin could enhance the effect of oxaliplatin to inhibit the migration and invasion of HepG2 cells by promoting the expression of E-cadherin 92.

### Colon cancer

**Figure 4:**
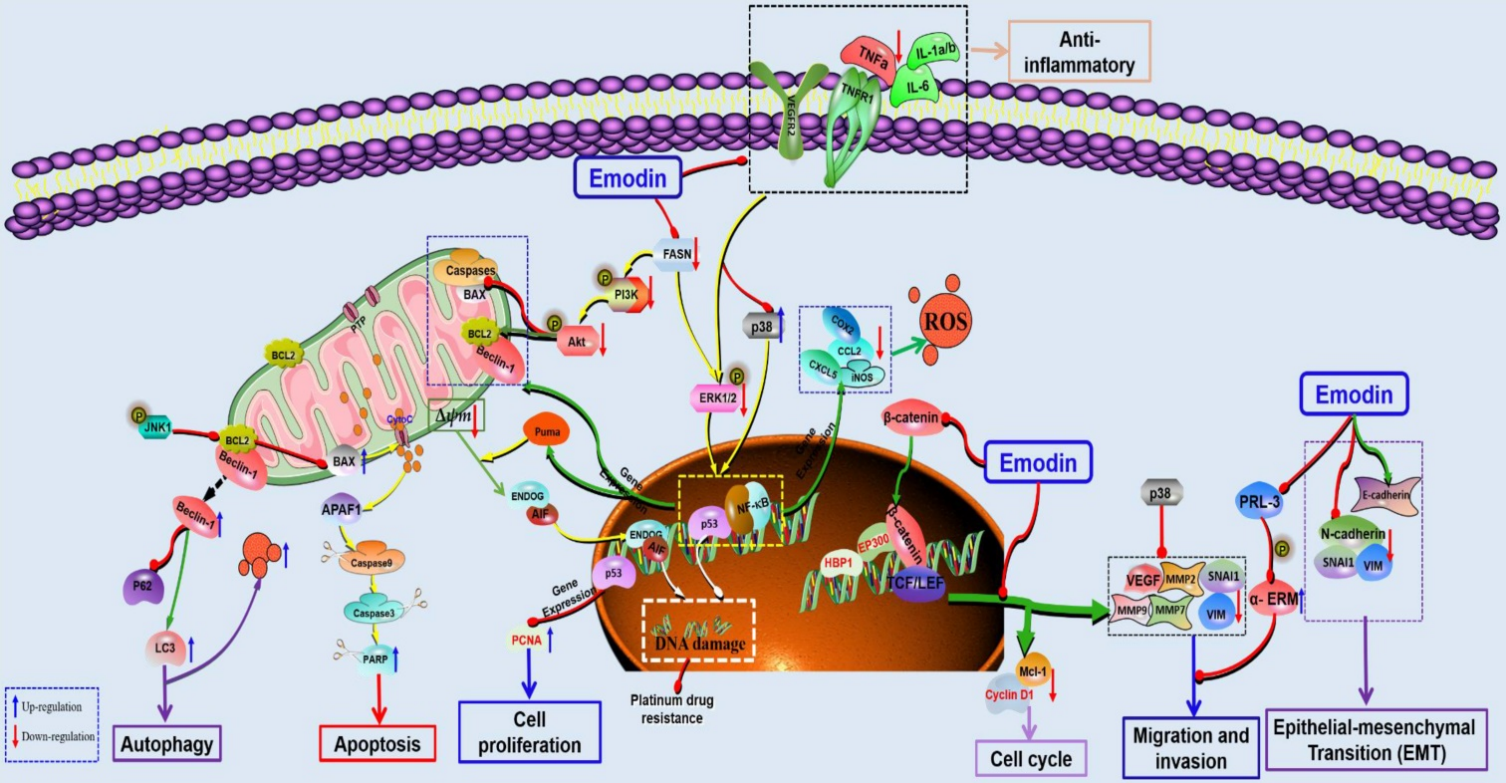
The potential mechanisms for antitumor effect of emodin against colorectal cancer.

Colorectal cancer (CRC) is another common cancer in China with high mortality and mortality 93-95. Increasing research has indicated that emodin is an effective natural agent for the treatment of CRC with promising prospects (Figure 4). In 2012, a study reported that emodin can induce G0/G1 phase arrest and mitochondria-mediated apoptosis in the colon cancer cell line LS1034 and inhibit the growth of xenograft tumors in nude mice *in vivo* 96. Another study revealed that the antitumor effect of emodin might be related to mitochondria-mediated apoptosis by regulating oxidative stress and p38/p53/Puma 97-99. Fatty acid synthase (FASN), a key enzyme for fatty acid synthesis, is highly expressed in tumor tissues of CRC patients, so it is considered a promising target for CRC treatment. In 2017, Lee *et al*. found that emodin had antiproliferation and proapoptotic effects on HCT116 cells by inhibiting intracellular FASN enzyme activity and reducing intracellular free fatty acids 100. The PI3K/Akt pathway is involved in the regulation of cell proliferation, differentiation, apoptosis, glucose transport and other physiological functions and has become a major focus of medical attention. A study in 2018 reported that the antitumor effects of emodin against the CRC cell line Caco-2 might be related to regulating PI3K/Akt 101. In addition, emodin can reverse the drug resistance of 5-FU-resistant CRC by regulating the PI3K/Akt pathway 102. Wang *et al*. reported that emodin can induce autophagy to promote the apoptosis of CRC cells and emphasized that autophagy is a necessary condition for CRC apoptosis via ROS accumulation 103.

Han et al. found that emodin could inhibit the migration and invasion of DLD-1 cells by inhibiting the activity of PRL-3 phosphatase 104. The ability of tumor cells to invade and migrate allows them to separate from the tumor tissue and enter the fluid circulation, which is the main cause of cancer metastasis and the main problem encountered in clinical treatment. Increasing evidence has suggested that epithelial-mesenchymal transformation (EMT) is essential for the invasion and metastasis of cancer cells. EMT can be activated by a variety of signaling pathways, among which Wnt/β-catenin signaling is an important pathway. Previous studies have suggested that emodin can regulate EMT via the Wnt/β-catenin pathway and inhibit the proliferation and growth of CRC cells. Emodin can downregulate key regulatory factors (such as β-catenin and TCF7L2) and their downstream targets (including cyclin D1, c-Myc, snail, vimentin, MMP-2 and MMP-9) in the Wnt signaling pathway to inhibit the migration and growth of the CRC cell lines SW480 and SW620. In addition, ROS play a key role in emodin-mediated downregulation of Wnt signaling 105. Gu *et al*. found that emodin can inhibit the invasion and migration of CRC cells* in vivo* and *in vitro* via downregulation of VEGF, MMP-7 and MMP-9 in tumor cells. Further studies found that emodin inhibited EMT through inactivation of Wnt/β-catenin signaling 106. Dai *et al*. reported that downregulation of VEGFR2 is also an important mechanism for the inhibitory effects of emodin against adhesion and migration in CRC 107. In addition, Zhang *et al*. found that anti-inflammation is also important for the antitumor effects of emodin against CRC. Emodin can inhibit the recruitment of inflammatory cells in the tumor microenvironment, such as CD11b^+^ and F4/80^+^, reduce cytokines, such as TNFα, IL1α/β, IL6, CCL2, and CXCL5, and inhibit COX-2 and NOS2 to inhibit the active adhesion, migration and invasion of CRC cells 108.

### Breast cancer

Breast cancer (BC) is one of the most common types of cancer in women and the second leading cause of cancer deaths worldwide 109, 110. Zhang *et al*. found that emodin could act as a tyrosine kinase inhibitor to inhibit the activity of HER-2/neu tyrosine kinase in MDA-MB453 cells, inhibit the growth of cancer cells, induce the production of lipid droplets, and promote the mature differentiation of BC cells 111. Furthermore, they also found that emodin can inhibit the transformation phenotype and metastatic ability of BC cells with HER-2/neu overexpression 112. In addition, it has been reported that the combination of emodin and paclitaxel can synergistically inhibit the growth and survival of BC cells, increase tumor sensitivity to paclitaxel, and improve tumor drug resistance. The mechanism is related to the reduction of tyrosine phosphorylation of HER-2/neu, suggesting that HER-2/neu inhibition is one of the important approaches of emodin in BC treatment 113. Ueno *et al*. found that emodin also effectively inhibited the growth of MDA-MB-435 cells with low HER-2/neu expression by inhibiting tyrosine kinase 114. In 2008, Huang *et al*. found that emodin could inhibit the proliferation of BCAP-37 cells and induce cell apoptosis 115, and subsequent studies revealed that emodin regulated 30 specific genes in BCAP-37 cells using an apoptosis-associated cDNA microarray, especially p53 and IGF-2 116. Li *et al*. found that emodin inhibited the growth of MCF-7 cells (IC_50_ = 7.22 μg/mL) and induced obvious apoptotic characteristics such as DNA fragmentation via both endogenous and exogenous apoptosis pathways 117.

Breast cancer is an estrogen-dependent malignant tumor that is closely related to estrogen activity, and estrogen needs to bind specifically with estrogen receptor (ER) to form a hormone-receptor complex to exert its biological effect. ERα is an important type of estrogen receptor in the body that is located in the nucleus and mediates the genotype effect of estrogen, that is, by regulating the transcription of specific target genes to exert a regulatory effect. Sui *et al*. found that emodin inhibited estrogen-induced proliferation of MCF-7 and MDA-MB-231 cells, promoted apoptosis and arrested the cell cycle in G0/G1 phase by downregulating the expression of cyclin D1 Bcl-2 and ERα proteins 118. Zu *et al*. also suggested that emodin has good antitumor effects on the BC cell lines BCAP-37 and ZR-75-30 119. Using virtual screening, Zhang *et al*. found that emodin is an effective aromatic hydrocarbon receptor (AhR) agonist. Subsequent *in vitro* experiments also found that the expression levels of AhR and cytochrome P450 1A1 (CYP1A1) in MCF-7 cells were significantly upregulated by emodin treatment, suggesting that the antitumor effects of emodin against BC might be related to the AhR-CYP1A1 signaling pathway 120.

Multidrug resistance to chemotherapeutic drugs is the main cause of BC treatment failure, and overexpressed ERCC1, a key protein in nucleotide excision repair (NER), is one of the main causes of drug resistance 121, 122. It was reported that emodin at 10 μg/mL could downregulate ERCC1 and inhibit doxorubicin (DOX)-cisplatin resistance in MCF-7 cells 123. In 2018, emodin (20 μM) was reported to increase the sensitivity of MCF-7 breast cancer cells to chemotherapy and promote 5-FU-induced apoptosis and senescence of BC cells. The mechanism was related to the inhibition of NRARP, and silencing NRARP blocked the effect of emodin on MCF-7 cells 124. DOX is a commonly used chemical drug against BC, but the rapid emergence of drug resistance is a major culprit limiting its clinical use. Li *et al*. reported that DOX combined with emodin can improve the sensitivity of MDA-MB-231 and MCF-7 cells to chemotherapy, and the mechanism is closely related to increasing γH2A in cancer cells and regulating AKT1-mediated DNA damage 125.

The adenosine 5ʹ-triphosphate (ATP)-gated Ca^2+^-permeable channel P2X7 receptor (P2X7R) is highly expressed in many tumors and cancer cells and has been found to play an important role in the migration and invasion of metastatic tumor cells. Jelassi *et al*. reported that emodin could significantly inhibit ATP-induced Ca^2+^ increase, specifically inhibit P2x7R-mediated currents, and inhibit the invasion ability of cancer cells *in vitro*, and these effects almost disappeared after P2x7R interference. In addition, emodin can inhibit the invasion of MDA-MB-435S cells overexpressing P2X7R in zebrafish, suggesting that emodin can specifically antagonize P2X7R to inhibit the invasion of BC cells 126. Solid tumor tissue is composed not only of tumor cells but also of a variety of nontumor cells, such as fibroblasts, adipocytes, endothelial pericytes, mesenchymal stem cells (MSCs) and immune cells, which together constitute the tumor microenvironment. The interaction between tumor cells and nontumor cells plays an important role in tumor progression and therapy 127, 128. Macrophages are the most abundant immune cells in primary and metastatic tumor tissues and play an important role in the genesis, development and metastasis of tumors. Tumor cells can recruit macrophages and secrete chemokines and growth factors to induce macrophages to produce an M2-like phenotype. Similarly, macrophages promote tumor growth by promoting angiogenesis, suppressing immune responses, regulating the extracellular matrix and promoting tumor cell migration. In 2014, it was reported that emodin can have an antitumor effect against BC by influencing the physiological activity of macrophages. The results showed that emodin can inhibit primary tumor growth, lung metastasis and lung macrophage infiltration in mice with orthotopic inoculation of 4T1 or EO771 BC cells 129. Recently, it was reported that emodin can inhibit the production of TGF-β1 in BC cells and macrophages, attenuate TGF-β1- or macrophage-induced EMT in BC cells and cancer stem cell (CSC) formation, inhibit the migration and invasion of BC cells, and prevent postoperative lung metastatic recurrence of BC 130. Iwanowycz *et al*. also suggested that emodin could inhibit tumor growth by inhibiting macrophage infiltration and M2-like polarization while increasing T-cell activation and reducing tumor angiogenesis. However, the tumor suppressive effect of emodin disappeared in tumor-bearing mice with macrophage deficiency. In addition, emodin can inhibit the migration and adhesion of macrophages to the tumor site by inhibiting the secretion of MCP1 and CSF1 and the expression of THY-1 in tumor cells, suggesting that emodin can act on BC cells and macrophages simultaneously, effectively blocking the feedback loop between the two cells and exerting an antitumor effect 131. Fibroblasts are also important parts of the tumor microenvironment, which can promote the remodeling of the extracellular matrix and the production of generated growth factors and cytokines (such as TGF-β), promote the growth and migration of tumor cells, and generate EMT phenotypes. Hsu et al. found that emodin could effectively inhibit EMT and migration of BT20 cells induced by fibroblasts, while emodin pretreatment could also significantly inhibit EMT and migration of BT20 cells induced by TGF-β 132. These results suggest that emodin plays an anticancer role against BC by improving the tumor microenvironment. In addition, Sun *et al*. also found that emodin could inhibit lung metastasis in MDA-MB-231 xenograft mice. Using the MDA-MB-231 cell model, it was found that the antitumor effects of emodin on BC metastasis were closely related to the downregulation of MMP-2, MMP-9, uPA, and uPAR and the decreased activity of P38 and ERK_1/2_ 133. Triple-negative breast cancer (TNBC) has the lowest survival rate among all BC subtypes due to its strong aggressiveness and metastasis. Previous studies have shown that peritumoral adipose tissue contributes to the invasion and proliferation of TNBC cells, and emodin could downregulate CCL5 and inhibit the growth and invasion of TNBC cell lines MDA-MB-231 and MDA-MB-453 134.

**Figure 5:**
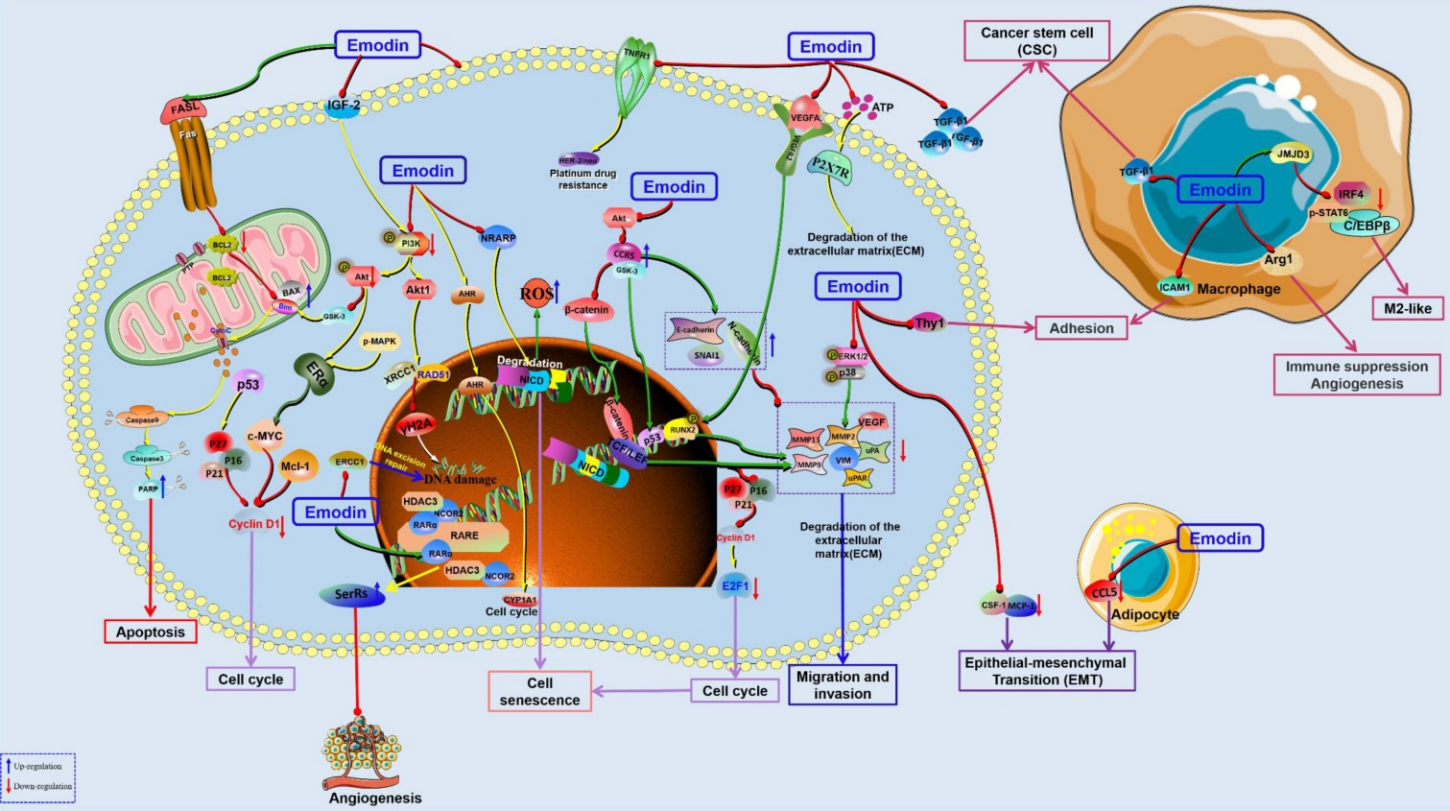
The potential mechanisms for antitumor effect of emodin against breast cancer.

Furthermore, another study reported that emodin inhibited BC cell-induced metastasis and angiogenesis by inhibiting the expression of MMPs and VEGFR-2. In addition, the antitumor effect of emodin against BC may be related to the downregulation of Runx2 transcriptional activity 135. Zou *et al*. found that emodin increased the expression of SerRS, which is a strong transcriptional inhibitor of VEGFA in TNBC cells. In addition, they identified a direct target of emodin, namely, nuclear receptor corepressor 2 (NCOR2). When NCOR2 binds to emodin, it is released from the SerRS promoter, resulting in activation of SerRS and ultimately inhibition of VEGFA transcription 136. In addition, many studies on BC are not limited to emodin alone but also include berberine, thymoquinone, daunorubicin, curcumin and other small molecule compounds. These combined therapies seem to achieve better antitumor effects, which may become an effective strategy for breast cancer treatment 137-140 (Figure 5).

### Pancreatic cancer

Pancreatic cancer (PC) is a malignant tumor occurring in the pancreatic exocrine gland and is one of the most common malignant tumors worldwide with high mortality 141-144. In addition to surgery, chemotherapy is another predominant way to improve the survival of patients with advanced PC, but drug resistance and side effects limit the clinical efficacy of the currently used chemotherapeutic drugs. Interestingly, an increasing number of studies have suggested that emodin might be a potential new drug for treating PC with less drug resistance and fewer side effects. It has been reported that the antitumor effects of emodin on PC growth may be related to the demethylation of tumor suppressor genes 145, 146. High expression of HIF-1α in tumor cells supports growth, angiogenesis, and high glycolysis, which is also known as the Warburg effect in tumors 147. Emodin can reduce the biosynthesis of HIF-1α in ASPC-1, BXPC-3, HPAF-2, MiaPaCa2, and PANC-1 cells and reduce their gene transcription or protein stability. In addition, the expression of HIF-1α-regulated downstream proteins (Glut1, HK-II, PFK-1, VEGF, caveolin-1, *etc*.) was also decreased, and emodin inhibited the phosphorylation of Akt and ERK/_1/2_ and downregulated intracellular signaling to reduce HIF-1α levels and attenuate cancer cachexia in athymic mice carrying cancer cells 148.

In 2008, Cai *et al*. reported the antitumor effect of emodin against PC, and emodin significantly inhibited the proliferation of four PC cell lines (Mia PacA-2, BXPC-3 panc-1 and L3.6PL) by inducing apoptosis 149. EGFR is overexpressed in 90% of pancreatic tumors, and EGFR-targeting drugs have become a hotspot in recent years. However, the drug resistance of targeted drugs seriously limits their clinical application 150-152. Interestingly, emodin was reported to increase the anti-proliferative effect of an EGFR inhibitor (afatinib) against PC through downregulation of EGFR by promoting STAT3 phosphorylation 153. Survivin, a member of the apoptosis suppressor gene family, is involved in controlling cell division and inhibiting apoptosis and is a target gene of β-catenin/Tcf/Lef. Guo *et al*. found that emodin enhanced the antitumor effect of gemcitabine against PC by downregulating survivin and β-catenin expression and reversed the drug resistance behavior of drug-resistant cells 154. In 2010, Liu *et al*. also reported that emodin enhanced the antitumor effect of gemcitabine on PC by downregulating NF-κB 155. Subsequently, they found that the synergistic effect of emodin on gemcitabine was associated with a decrease in Bcl-2/Bax and promotion of Cyt-C release from mitochondria to the cytoplasm 156. In addition, chemotherapeutic resistance to gemcitabine has also been reported to be associated with Akt activation, and gemcitabine combined with emodin increased cell death and mitochondrial fragmentation by inhibiting Akt phosphorylation and increasing the activation of caspase-3 and -9 157. XIAP is another important member of the anti-apoptotic gene family, which is highly expressed in many tumor cells and promotes tumor cell proliferation and anti-apoptosis. Wang *et al*. found that gemcitabine intervention upregulated XIAP expression in SW1990 cells and PANCC-1 cells, which was associated with the development of clinical drug resistance. Interestingly, the addition of emodin can significantly downregulate XIAP 158, 159. In addition, the combination of emodin can also reverse the resistance of BXPC-3 and SW1990 cells to gemcitabine by reducing the function of multidrug resistance gene-1 (MDR-1), reducing the expression of transmembrane glycoprotein P-gp (which pumps chemotherapeutic drugs out of cells), activating the mitochondrial apoptosis pathway, and reducing the resistance of BXPC-3 and SW1990 cells to gemcitabine 160, 161. Guo *et al*. reported that emodin can enhance the antitumor effect of gemcitabine (the gold standard chemotherapy drug for PC) by inhibiting MDR1/P glycoprotein, MRP expression, and the IKKβ/NF-κB signaling pathway 162. A recent study also found that emodin reversed gemcitabine resistance in PC cells by inhibiting the IKKβ/NF-κB signaling pathway 163.

**Figure 6:**
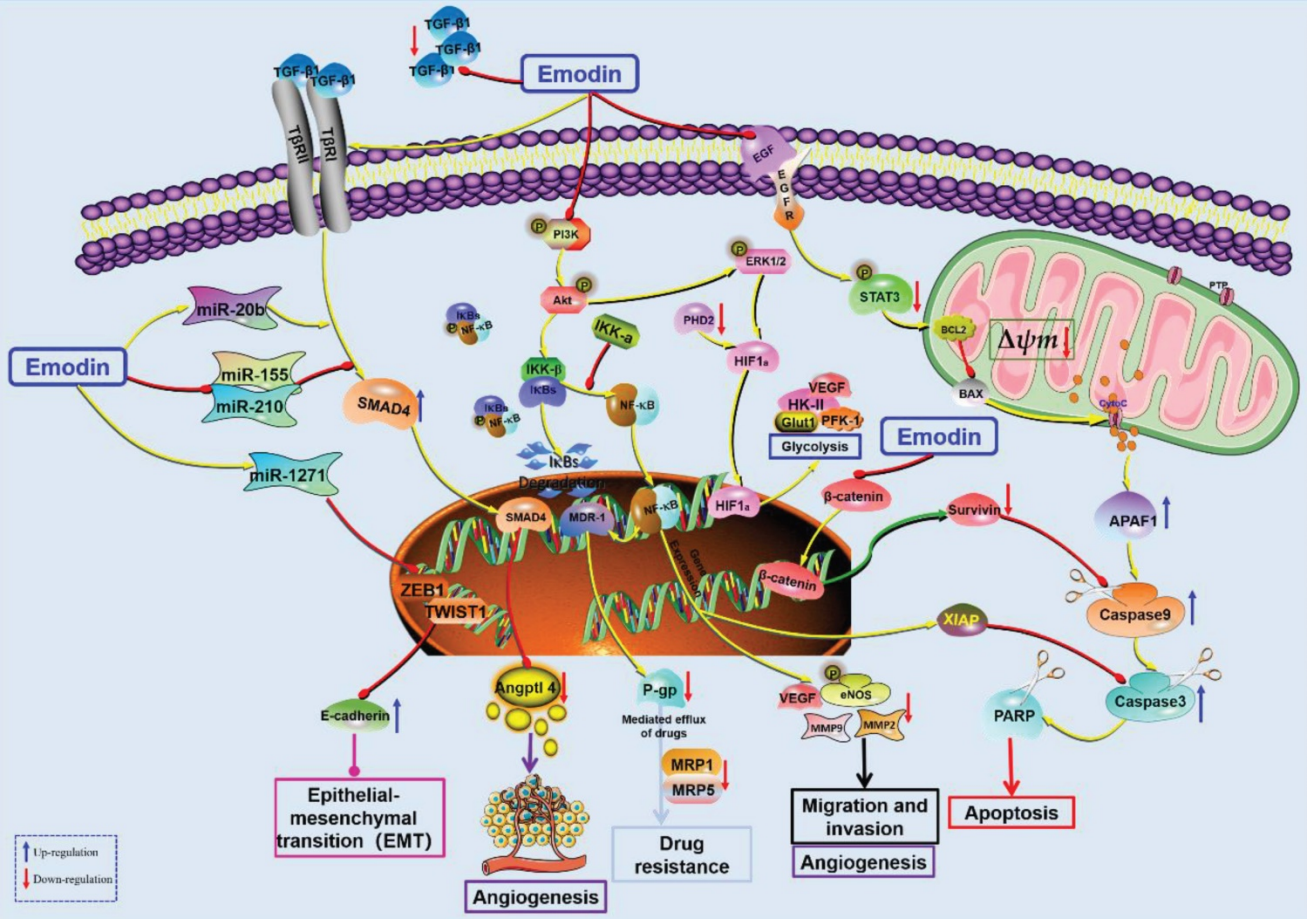
The potential mechanisms for antitumor effect of emodin against pancreatic cancer.

In addition, emodin has been reported to have significant anti-proliferation and anti-metastasis effects on PC by downregulating NF-κB DNA-binding activity and survivin and MMP-9 in PC cells and promoting apoptosis 164, 165. Li *et al*. found that emodin could significantly inhibit the proliferation, migration and invasion of SW1990* in vitro* and regulate the expression of nuclear genes encoding EMT-related proteins. It has also been found that emodin can inhibit hepatic metastasis of PC *in vivo* by inhibiting EMT and invasion of PC based on the ug-regulation of miR-1271 166. Further investigations found that emodin can inhibit tumor angiogenesis in PC and reduce the expression of the angiogenesis-related factors NF-κB, VEGF, MMP-2 and eNOS and the phosphorylation of eNOS 167. In addition, emodin inhibited angiogenesis in tumor tissues by altering the TGF-β/Smad pathway and the activities of angiogenesis-related miR-20b, miR-155 and miR-210 168 (Figure 6).

### Leukemia

Recently, accumulating studies have shown that emodin has obvious antitumor effects against chronic myelogenous leukemia (CML). CML is a common malignant tumor characterized by abnormal proliferation and accumulation of mature granulocytes 169. It was first discovered in 1973 that CML in more than 90% of patients is caused by a genetic variation called the Philadelphia (Ph) chromosome 170. Brown et al. found that a 100-fold reduction in the dose of Arsenic trioxide (As_2_O_3_) in combination with emodin could be highly toxic to APL tumor cells 171. In 2002, Chen et al. studied the anti-CML effect of emodin for the first time and found that emodin exhibited significant cytotoxicity to HL-60 cells, accompanied by the emergence of a DNA ladder. Further studies showed that emodin could induce apoptosis of HL-60 cells by activating the caspase cascade, but this antitumor activity was independent of ROS production 172-176. Emodin also showed antitumor effects in U937 cells via a decrease in Bcl-2/Bax and activation of pro-caspase-3 177. Myeloid cell leukemia 1 (Mcl-1), a signal transducer and activator of transcription 3 (STAT3)-regulated molecule, is necessary for myeloma cell survival. In 2007, Muto *et al*. found that emodin inhibited the growth of multiple myeloma (MM) cells and induced cell apoptosis by inhibiting Janus-activated kinase 2 (JAK2) activity and phosphorylation of STAT3 and downregulating Mcl-1 178. Zheng et al. confirmed that the anti-proliferation, cell cycle arrest and apoptosis induced by emodin in HL-60 cells were related to the inhibition of the Akt pathway 179. Chen *et al*. reported that emodin inhibited proliferation and cell colony formation and induced cell apoptosis due to G0/G1 phase arrest in HL-60/ADR cells 180. Importantly, Chen et al. further indicated that emodin, as a novel PI3K/Akt inhibitor, can specifically inhibit the phosphorylation at tSer473 of Akt and Ser2448 of mTOR in acute myeloid leukemia (AML) cells 181. In addition, emodin-induced apoptosis in K562 cells was also associated with the inhibition of PETN/PI3K/Akt and BCR-ABL deletion 182. In addition, endoplasmic reticulum stress (ERS), the caspase cascade and independent mitochondrial pathways are also involved in emodin-induced apoptosis of the leukemia cell line WEHI-3 183.

**Figure 7:**
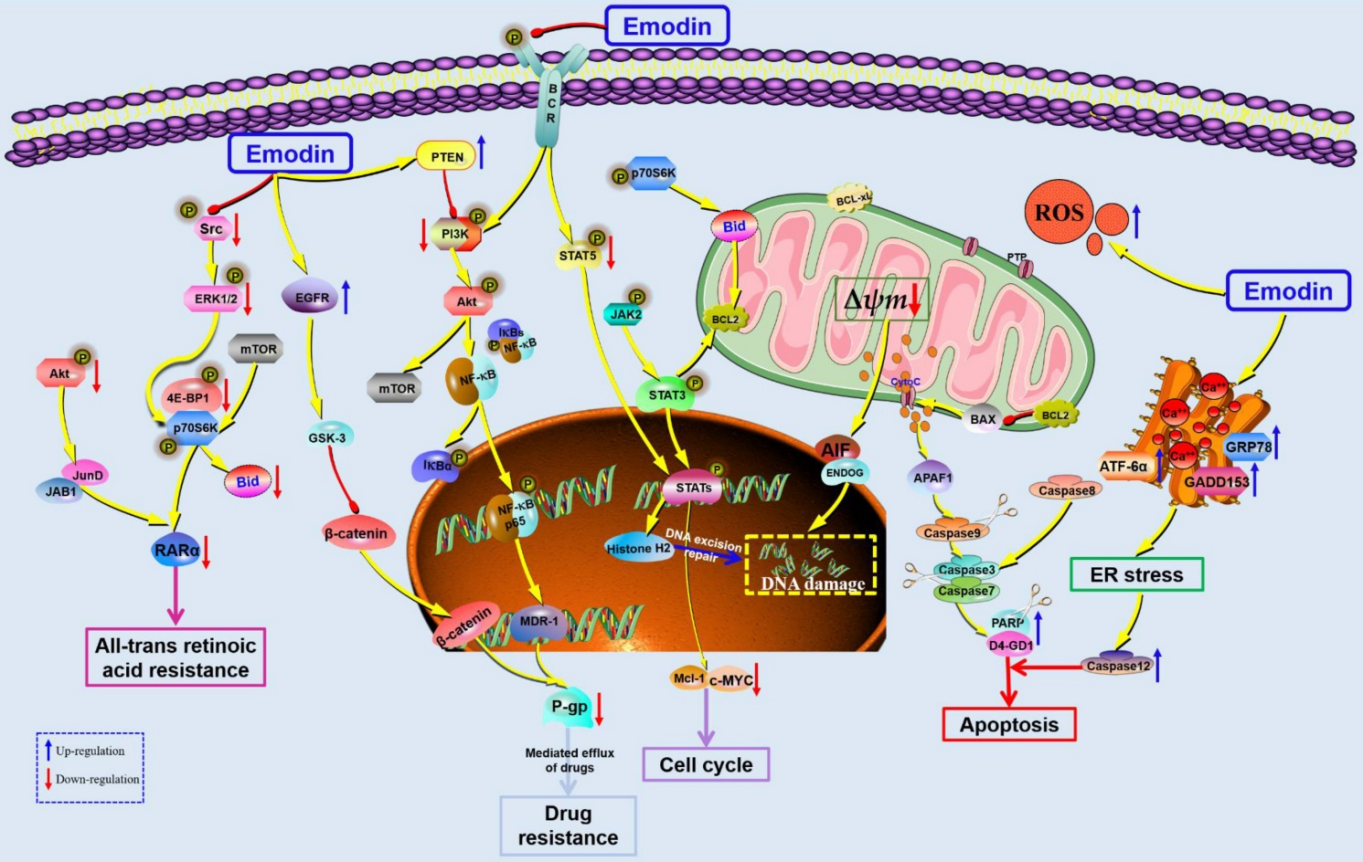
The potential mechanisms for antitumor effect of emodin against Leukemia.

The 3'-azido-3'-deoxythymidine (AZT) can competitively inhibit the reverse transcription process, inhibit telomerase activity, block telomere elongation, and inhibit the growth of cancer cells 184-186. It has been reported that P-gp is also highly expressed in the tumor tissues of CML patients, leading to drug resistance. Interestingly, emodin combined with AZT can synergistically induce the growth of K562/ADM cells and S-phase cell arrest by downregulating MDR1 and P-gp 187. Furthermore, Min et al. found that emodin may be a potential substrate of P-gp with competitive binding at its R site 188. Another study reported the synergistic effect of emodin on AZT through upregulation of early growth response-1 (EGR1) and inhibition of the Wnt/β-catenin pathway 189. In addition, emodin combined with cytarabine (ARA-C) can significantly inhibit the growth of HL-60/ADR cells *in vivo* and *in vitro* through dual targeting of the Akt and ERK pathways 190. A recent study reported that emodin can enhance the sensitivity of K562/G01 cells to imatinib via inhibition of phosphorylation of Bcr-Abl and STAT5 and phosphorylation of Src 191 (Figure 7).

### Gynecological cancer

#### Cervical cancer

As the second most common malignant tumor in females in the world, cervical cancer seriously threatens the lives and health of women. Human papillomavirus (HPV) infection has been identified as the main cause of cervical cancer, especially HPV16 and HPV18. Type I interferons (IFN-α and IFN-β) play important roles in innate immune responses and can play antiviral, antitumor, antiproliferative and immunomodulatory roles by activating JAK/STAT pathway signals 192. The 26S proteasome is a molecular complex that catalyzes ubiquitination protein degradation and is also a negative regulator of IFN-α/β signal transduction, participating in the negative regulation of JAK/STAT. Inhibition of the 26S proteasome to enhance the immunomodulatory effect of IFN-α/β is a new strategy for the development of antitumor drugs. Emodin was found to be an effective inhibitor of the human 26S proteasome, which increased phosphorylation of STAT1, decreased phosphorylation of STAT3, increased endogenous gene expression stimulated by IFN-α, inhibited the degradation of type I interferon receptor 1 (IFNAR1), and enhanced the anti-proliferation effect of IFN-α on HeLa cervical cancer cells 193. In 2003, Srinivas *et al*. reported the anti-proliferative effect of emodin on cervical cancer cells via the induction of apoptosis 194. Furthermore, Olsen *et al*. suggested that the antitumor effect of emodin against cervical cancer might be related to regulation of the PI3K/Akt pathway 195. In 2013, emodin was reported to induce HeLa cell apoptosis through endogenous mitochondrial and exogenous death receptor pathways via regulation of caspase-8 196. Wang *et al*. found that the antitumor effects of emodin against cervical cancer (HeLa) might be related to many mechanisms, such as apoptosis, autophagy, G0/G1 phase arrest and angiogenesis inhibition 197.

In 2017, Trybus *et al*. found that emodin could cause significant changes in the lysosomal compartment of HeLa cells, which manifested as an increase in the number of lysosomes and an increase in autophagic vacuoles, leading to lysosomal membrane damage 198. Furthermore, a recent study reported that emodin is a photosensitizer in combination with photodynamic therapy that significantly increased cytotoxicity to SiHa, CaSki, and HaCaT cells, which was associated with increased ROS production, caspase-3 activity, and autophagy 199. The TGF-β signaling pathway is involved in the growth, development, and differentiation of tumor cells and can also induce epithelial cells to transform into mesenchymal cells, promoting advanced tumor progression. It has been suggested that emodin can regulate TGF-β signaling in SiHa and HeLa cells and affect the growth, migration, and invasion of cervical cancer cells via downregulation of the TGF-β signaling pathway by reducing TGF-β receptor II and Smad4, inhibiting cyclin D1, P21 and Pin1, and downregulating Snail and Slug 200.

#### Ovarian cancer

Ovarian cancer is another common gynecological cancer worldwide, causing 152,000 cancer-related deaths each year 201. In 2009, Kurokawa et al. reported that emodin enhanced the toxicity of cisplatin against the human ovarian cancer cell line A2780, and this effect of emodin could be eliminated by knocking down hCtr1 202. In 2018, Song *et al*. reported a novel mechanism of emodin against ovarian cancer by promoting FOXD3 and activating miR-199a in turn, which inhibited TGF-β2 and reduced A2780 cell viability and colony formation 203. Emodin can induce apoptosis and inhibit cell invasion in SKOV3 and HO8910 cells by regulating survival 204. In addition, emodin can inhibit the proliferation, invasion and migration of ovarian cancer cells via suppression of EMT by regulating the ILK/GSK-3β/Slug and GSK-3β/β-catenin/ZEB1 signaling pathways 205, 206. A subsequent study further confirmed the mentioned effects of emodin on ovarian cancer cells without targeted toxicity to hepatocytes, renal cells and cardiomyocytes in nude mice 207.

Ovarian cancer cells, like other malignancies, are resistant to anticancer drugs. Interestingly, emodin can overcome paclitaxel resistance in A2780 cells by downregulating P-gp, XIAP and survival 208. In addition, emodin can also assist in enhancing the anticancer effects of cisplatin by inducing ROS production and downregulating MRP1 209. A recent study found that emodin can be recognized as an inhibitor of Aurora kinase A (AURKA) kinase and induce apoptosis in cisplatin-resistant ovarian cancer cells 210.

### Others

#### Head and neck cancer

Some studies have also reported antitumor effects of emodin on head and neck cancers. Emodin can induce apoptosis and cell cycle arrest in human tongue carcinoma SCC-4 cells via regulation of many proteins, such as P21, Chk2, cyclin B1, CDC2, cyto-C, caspase-3, -9, Bcl-2, Bax, GADD153 and GRP78 211. Chen et al. found that emodin can induce DNA damage in SCC-4 cells by inhibiting DNA damage repair genes, such as ataxia telangiectasia mutated (ATM), ataxia-telangiectasia and Rad3-related (ATR), 14-3-3sigma (14-3-3σ), breast cancer 1 early onset (BRCA1), and DNA-dependent serine/threonine protein kinase (DNA-PK) 212. In addition, another paper by Chen et al. found that emodin could inhibit the migration and invasion of SCC-4 cells by inhibiting MMP-2, U-PA, FAK, NF-κB P65, pAKT, P-P38, P-JNK, P-ERK, and MMP9 and promoting TIMP-1 213.

Similarly, emodin treatment can also induce morphological changes and rapid apoptosis of EC-109 cells by inducing intracellular ROS eruption and significantly reducing pH 214. The ectopic expression of TWIST1 in head and neck squamous cell carcinoma cells triggers EMT and leads to the acquisition of mesenchymal phenotypes of tumor cells, while the increased proportion of CD44-labeled cells in the tumor population can activate tumor initiation. Emodin treatment reduced the tumor-initiating ability of FaDu-pFLAG-TWIST1 cells and inhibited cell migration and invasion by inhibiting the β-catenin and Akt pathways to inhibit TWIST1-induced EMT 215. In another study, emodin significantly inhibited the growth and proliferation behavior of Tca8113 cells. In addition, emodin also caused G0/G1 phase arrest in Tca8113 cells and reduced the expression of CDK2, Cyclin E and P21 216. Furthermore, emodin also inhibited oral tumorigenesis in DMBA-treated hamsters by regulating cell cycle markers such as cyclin D1, PCNA, CDK4, CDK6 and survivin 217, and the proapoptotic and antioxidant effects of emodin also play important roles in this process 218. Moreira *et al*. reported that emodin plays an antitumor role in oral squamous cell carcinoma cells (HSC-3) by increasing oxidative stress and DNA damage, inhibiting Akt activation, and activating apoptosis and necrosis 219.

A similar anticancer effect of emodin has been observed in human nasopharyngeal carcinoma cells. Emodin can significantly inhibit the growth of CNE-2Z cells and induce their cycle arrest and apoptosis 220.

TNF receptor-associated factor 6 (TRAF6) is closely associated with tumor angiogenesis and metastasis. Emodin can inhibit angiogenesis and metastasis in anaplastic thyroid cancer (ATC) by inhibiting the TRAF6/HIF-1α/VEGF and TRAF6/CD147/MMP9 signaling pathways 221. In addition, it has also been reported that the antitumor effect of emodin on thyroid papillary carcinoma is related to the activation of AMPK and inhibition of the MEK-ERK pathway 222.

#### Glioma and Neuroblastoma

In 2005, Kim *et al*. found that emodin can effectively inhibit HA-induced MMP9 secretion and invasion of glioma by inhibiting the activation of FAK, ERK_1/2_ and Akt/PKB, as well as the transcriptional activities of AP-1 and NF-κB 223. In glioblastoma (GBM) cells treated with ionizing radiation (IR), fructose 1,6-bisphosphatase 1 (FBP1) is downregulated, along with increased glucose uptake and extracellular acidification, indicating increased intracellular glycolysis. At the same time, intracellular Ets1 was overexpressed, suggesting that Ets1 is a transcriptional inhibitor of FBP1. Emodin inhibited the glycolysis rate and IR-induced GBM migration in orthotopic xenograft mice 224. Kim *et al*. evaluated the effects of emodin on glioma stem cells (GSCs) and found that emodin can significantly inhibit the self-renewal activity of GSCs, partially induce apoptosis, inhibit cell invasion, and increase the sensitivity of GSCs to IR. In addition, emodin can promote EGFR/EGFRvIII degradation by blocking the interaction between EGFR/EGFRvIII and Hsp90 and inhibiting the expression of Notch, β-catenin and STAT3, which leads to the inhibition of GSC stemness 225. Another study reported that emodin can inhibit proliferation and induce apoptosis and necrosis in U251 cells by upregulating TNF-α, RIP1, RIP3 and MLKL levels and activating the TNF-α/RIP1/RIP3 axis 226.

The antitumor effects of emodin have also been observed in neuroblastoma cells. When sh-SY5Y cells were exposed to emodin, cell viability was significantly decreased. In addition, emodin also significantly inhibited the migration and invasion of SH-SY5Y cells by inhibiting GRB2, RhoA, HIF-1A, VEGF, FAK, iNOS, COX2, p-P38, p-C-Jun, MMP2, MMP9 and MMP7 and promoting PKC, PI3K, MEKK3 and NF-κB p65 227. Emodin can induce apoptosis of human neuroblastoma IMR-32 cells by regulating ROS, P53, P21, caspase-3, -9 228.

#### Prostate cancer and bladder cancer

Prostate cancer is one of the most common malignant tumors of the male genitourinary system. The level of androgen in the body is closely related to the incidence of prostate cancer. Abnormal secretion of androgen is also the main cause of inducing male prostate cancer. Androgen receptor (AR) is involved in the effect of androgen on tumor initiation and plays a major role in the recurrence and outcome of prostate cancer. It has been reported that emodin can directly target AR to inhibit the growth of prostate cancer cells and prolong the survival time of tumor-bearing mice 229. Yu *et al*. observed that emodin could inhibit the proliferation of prostate cancer cells LNCaP via the mitochondrial apoptosis pathway by decreasing the expression of AR and PSA and increasing the expression of p53 and P21 230. Emodin can also increase the chemotherapy sensitivity of DU-145 cells to cisplatin and inhibit the growth of prostate tumors by inducing ROS production, downregulating MDR1, inhibiting HIF-1, and promoting drug retention 231. Masaldan et al. also confirmed that the inhibitory effect of emodin on the proliferation of LNCaP and PC-3 cells was largely related to inducing high levels of ROS production. In addition, they found that LRP1 also appears to play an important role in emodin's antitumor effects 232. Furthermore, the CXCR4/CXCL12 axis is reported to be involved in promoting tumor invasion and metastasis, and downregulation of CXCR4 is of great significance in inhibiting cancer metastasis. Emodin can inhibit the migration and invasion of DU145 cells by downregulating CXCR4 mRNA and inhibiting NF-κB activation 54. Another study found that emodin upregulated Notch1, promoted Notch1 nuclear transfer and inhibited Jagged1, VEGF and bFGF 233.

Epigenetic studies showed that histone H3K27 trimethylation (H3K27me3) expression was low in human bladder cancer, and histone H3S10 phosphorylation (pH3Ser10) expression was high, while the two epigenetic markers showed opposite expression patterns in normal bladder cancer. Emodin could inhibit pH3Ser10, FABP4 & HBP17 and elevate H3K27me3 234. Chemotherapeutic tolerance is one of the main causes of tumor progression and recurrence of bladder cancer, and ROS are a key factor in the chemical sensitivity of cancer cells. Emodin can effectively enhance the cytotoxicity of cisplatin-treated human bladder cancer cells T24 and J82 by increasing the ROS level through reducing the GSH-cisplatin conjugate. In line with *in vitro* experiments, emodin/cisplatin therapy also increased xenograft tumor cell apoptosis, and MDR1 was ultimately responsible for these phenomena 235.

#### Lymphoma

In 2013, emodin reduced the number of living cells in Dalton's lymphoma (DL) and significantly extended the lifespan of DL mice by inducing the production of H_2_O_2_ and reciprocal regulation of degradation antioxidant enzymes, inducing the DL cell mitochondrial apoptosis pathway 236. Lin *et al*. reported that emodin can reduce the survival rate of Raji cells via induction of cell apoptosis by downregulating ubiquitin-like protein containing PHD and RING domains 1 (UHRF1). In addition, they found that emodin could increase the sensitivity of Raji cells to adriamycin 237. To explore the mechanism of emodin in aggressive non-Hodgkin's lymphoma (NHL), Chen et al. predicted the potential anti-NHL targets of emodin with the help of bioinformatics techniques. The results showed that TP53 and PI3K may be important molecules and pathways of emodin in NHL treatment. Interestingly, subsequent experiments confirmed the predicted results that emodin could significantly inhibit the proliferation and induce apoptosis of SU-DHL4 cells, and these pharmacological effects were related to the inhibition of TP53 and phosphorylation of PI3K/Akt 238.

#### Gallbladder carcinoma

Gallbladder carcinoma (GBC) is one of the most common malignant tumors of the bile duct, ranking 6^th^ among gastrointestinal tumors worldwide 239, 240. However, GBC is resistant to many anticancer drugs, which is the main obstacle to its clinical treatment. Wang *et al*. found that emodin combined with cisplatin, carboplatin or oxaliplatin significantly enhanced the chemosensitivity of SGC996 cells, which was associated with the inhibition of glutathione levels and MRP1 in SGC996 cells. In addition, emodin combined with cisplatin inhibited tumor growth by increasing apoptosis and downregulating MRP1 241. Another study reported that emodin promotes cisplatin-induced apoptosis of GBC cells by inhibiting survivin 242.

#### Osteosarcoma

Qu et al. evaluated the effect of emodin on angiogenesis in human osteosarcoma and found that exogenous HMGB1 supplementation positively promoted angiogenesis in nude mouse grafted tumor tissues, as demonstrated by increased VEGF and vWF expression, which was effectively reversed by emodin treatment. *In vitro*, emodin also significantly inhibited the proliferation of the osteosarcoma cell lines SOSP-9607, MG63 and SAOS-2, decreased HMGB1-induced VEGF production, and increased the expression of SIRT1 and deacetylase activity 243. In another study, they evaluated the inhibitory effect of emodin on the antiradiation ability of MG63 cells. Emodin treatment inhibited cell viability and survival of MG63R cells after irradiation, as well as colony formation, and increased cell apoptosis. Further studies showed that emodin inhibited the expression of Shh and Bcl-2 and the nuclear translocation of Gli1 in MG63R cells after radiation exposure and increased the expression of C-caspase-3 244. Ying *et al*. found that emodin inhibited human osteosarcoma cells by ROS-independent ER stress 245. Similarly, emodin can enhance the antitumor effect of cisplatin in human osteosarcoma via the Nrf2 pathway 246.

#### Skin cancer

In 2002, emodin was reported to have a strong inhibitory effect on excessive nitric oxide (NO)-induced skin canceration in mice 247. It has been reported that emodin can increase ROS levels and induce apoptosis in mouse melanoma B16F10 cells 248. Emodin can significantly reduce the expression of CD155 in tumor cells, inhibit the proliferation and migration of tumor cells, and induce cell cycle arrest in the G2/M phase. Similarly, emodin has been observed to inhibit tumor growth and inhibit CD155 expression in B16 melanoma mice 249. Another study found that emodin acted as a mitochondrial decoupler, downregulating ATP levels in tumor cells and inhibiting growth 250. Li *et al*. found that emodin had significant antiproliferation and proapoptotic activities on B16F10 and A375 melanoma cells with strong metastatic ability and significantly inhibited the migration and invasion of tumor cells 251.

#### Gastric cancer

In addition, emodin also showed anticancer activity against gastric cancer. Cai et al. found that emodin, as an ROS producer, can combine with arsenic trioxide (ATO) to cause oxidative stress in SGC-7901 cells, promote RhoA inactivation, lead to actin silk breakage, and lead to structural destruction of the site adhesion complex, ultimately leading to anoikis, which can be partially reversed by the antioxidant N-acetylcysteine (NAC) 252. In addition, emodin can inhibit proliferation and induce apoptosis of SGC-7901 cells by downregulating prL-3 253.

## Metabolic transformation of emodin

It is of great significance for directional synthesis, structural modification and activity screening of drugs to explore the metabolic or biotransformation process and pathway of drugs *in vivo* and further confirm the structure of metabolites. The liver is considered to be the main site of metabolism, with over 50% of oral emodin found in bile 254. However, emodin seems to be more important in the gut. A 2010 study found that the maternal level of emodin in blood samples of rats decreased rapidly after intravenous injection of emodin, while the emodin glucuronides omega-hydroxy emodin (ω-OHE, a phase I metabolite) and ω-OHE sulfates/glucuronides immediately appeared. However, after oral administration of emodin, only emodin glucuronides were found in serum, while emodin, ω-OHE and ω-OHE sulfates/glucuronides were not detected, which may be because most emodin will be metabolized in the intestine first after absorption, and a small amount will reach the liver for stage I transformation. Furthermore, the results of this study clearly show that the intrinsic clearance values of emodin are very high, leading to the rate of emodin's glucuronidation being rapid via the liver and intestinal microsomes of male rats. This suggests that the oral bioavailability of emodin parents is almost zero due to rapid and extensive binding metabolism during delivery 255. Glucuronidation metabolism via glucuronidation in the gut appears to be one of the main reasons for the low bioavailability of emodin, and the other important reason is its low solubility. Therefore, reducing emodin glucuronidation and improving its solubility are effective means to improve its bioavailability. In addition, there was a sex difference in the rate of emodin glucuronidation in animals, which was due to the unique expression pattern of UGT2B1 that facilitated this process in male mice. However, because UGT2B1 is not expressed in humans, there may be no sex effect of emodin glucuronidation in humans 256. In addition, the absorption of emodin on Caco-2 cells showed significant concentration dependence. The higher the concentration was, the higher the rate of emodin absorption, indicating that emodin was mainly absorbed by passive diffusion in Caco-2 cells 257.

## Toxicity of emodin

Although emodin has excellent performance in the treatment of cancer, the toxicity or adverse reactions caused by long-term use should not be ignored. Radiac Brkanac et al. used human peripheral blood lymphocytes (HPBLs) to study the cytotoxicity, genotoxicity and oxidative stress parameters of emodin and found that emodin could induce cell death and DNA damage at concentrations of 150 μg/mL and 200 μg/mL. At 25 μg/mL, emodin induced an ROS increase, suggesting that emodin has cytotoxicity and genotoxicity against HPBLs, and oxidative stress is involved in its toxic mechanism 258. In addition, studies have reported that emodin can cause hepatotoxicity in rats by activating CYP3A and consuming GSH 259. In an experimental study conducted at the National Institutes of Health over two years, high doses of emodin (280, 830, 2500 ppm) were found to induce zymmbal adenocarcinoma in female F344/N rats but were not carcinogenic in female F344/N rats. Rare renal tubular neoplasms also occur in male B6C3F1 mice. However, low doses of emodin (312, 625, 1250 ppm) showed no carcinogenic effect, suggesting that emodin over a certain dose range has a certain carcinogenic effect, and this effect is different between genders 260. In addition, cytochrome P450 1A2 is involved in the formation of emodin metabolites in the liver 261. Similarly, Wang et al. also found that emodin can induce the expression of P450 1A1 and 1B1 in human lung adenocarcinoma CL5 cells, which may be an important factor affecting the metabolism and toxicity of emodin *in vivo* 262. Another study also described the embryotoxicity of emodin, with emodin ingestion leading to cell apoptosis and decreased cell proliferation, inhibiting early embryo development to the blastocyst stage. Meanwhile, 25-75 μM emodin could induce apoptosis of blastocyst cells and decrease the success rate of blastocyst implantation. In addition, *in vitro* treatment with emodin can cause fetal weight loss 263. A study in 2015 also reported the reproductive toxicity of emodin, which significantly inhibited the total motility, forward motility and linear velocity of sperm at levels greater than 100 μM. The mechanism may be related to a reduced intracellular Ca^2+^ concentration and tyrosine phosphorylation 264. A recent study evaluated the hepatorenal toxicity of emodin in KM mice and found that high doses of emodin (600 mg/kg) for 28 consecutive days produced significant systemic toxicity in mice, accompanied by liver, kidney, gallbladder and spleen injury 265. In conclusion, in addition to the wide range of activities of emodin, we should also pay attention to and correctly understand the toxic reactions caused by different doses of drugs.

## Conclusion

**Figure 8:**
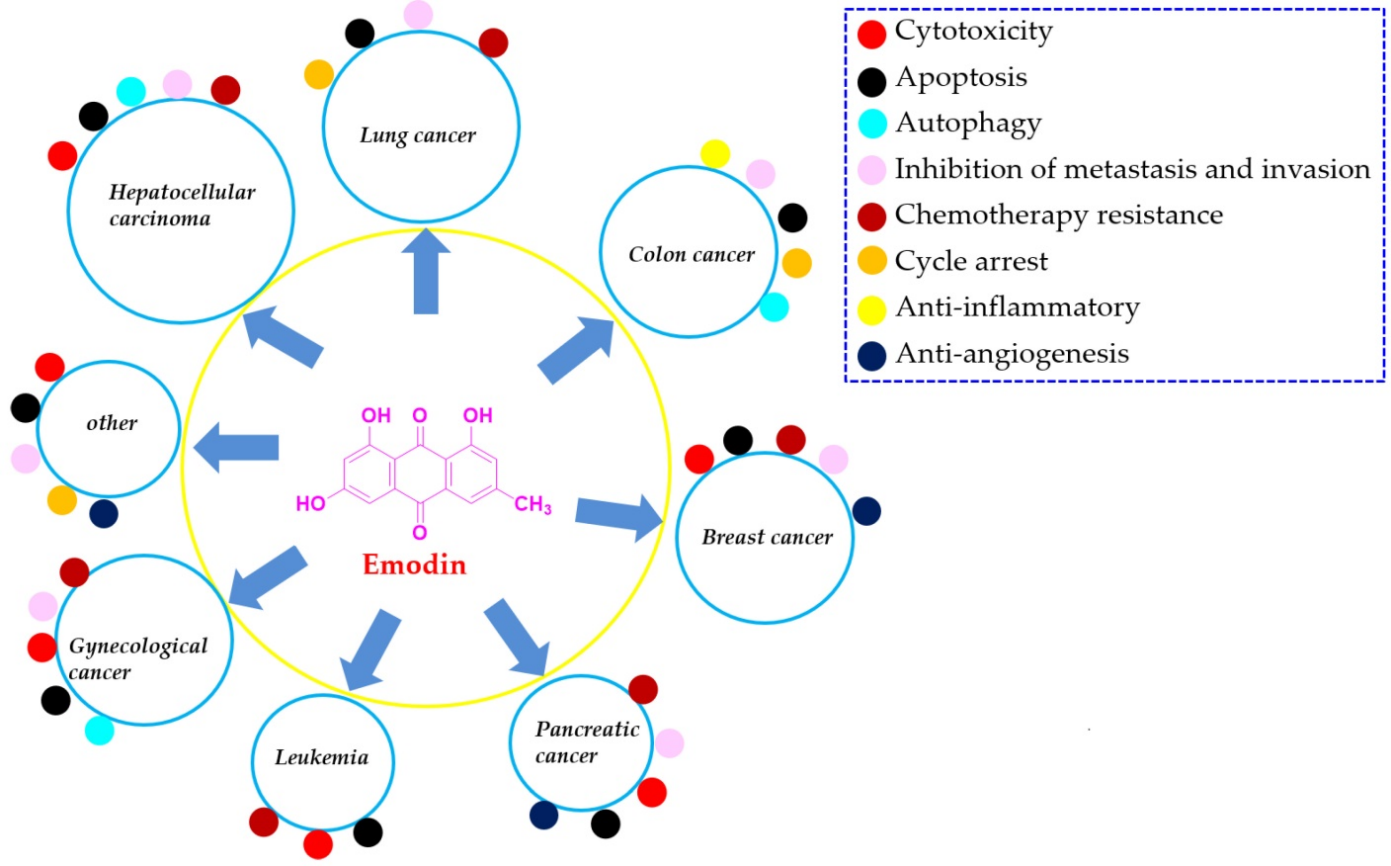
The main molecular mechanism of emodin anticancer action.

In recent decades, an increasing number of pharmacists have suggested that natural products are valuable resources for finding reliable candidate drugs 266,267. In this paper, we summarized the reported references that investigated the natural anthraquinone of emodin with versatile potential in the chemotherapy of various types of cancer, such as lung cancer, liver cancer, colon cancer, breast cancer, and pancreatic cancer. Currently, the continued progress made in various aspects of this natural agent suggests that emodin might be a promising lead compound for development as a new clinical chemotherapy drug for treating various cancers, especially lung cancer, liver cancer, and breast cancer (Figure 8).

However, there are still many areas in both preclinical and clinical investigations of emodin that need to be improved upon in the future. First, most of the presented studies about the antitumor effects of emodin are *in vitro* cell studies, and the animal evidence is inadequate. Therefore, more work should be devoted to animal studies to evaluate the antitumor effects of emodin *in vivo*. Second, the available data of this natural compound have primarily focused on preclinical studies but rarely clinical experiments for treating tumors. Therefore, future studies of emodin may devote more work to the study of its therapeutic effects in the clinic. Third, tumors commonly require long-term treatment, so the safety of the candidate drug is very important. However, few investigations of the target organ toxicity of emodin have been carried out thus far. Therefore, more drug safety studies might be encouraged to explore the side effects of emodin. Fourth, the bioavailability of emodin *in vivo* is also very low, which is an important limitation for the drug development of this monomer. Due to this limitation, chemical structure modification and novel nanodrug delivery systems for emodin might be promising strategies to solve this problem. Lastly, previous references mentioned lots of targets for emodin, however, the real drug targets for emodin are still needed to be further clarified due to most of the reported proteins regulated by emodin might be the signalling molecules instead of the drug targets. In conclusion, this paper provides an updated overview of the current studies regarding the antitumor effects of emodin, which is helpful for the development of this natural monomer as a candidate drug for treating cancers in the clinic.

## Figures and Tables

**Table 1:** Antitumor potentials of emodin

Cancers	Mechanisms	Cell lines/Animals	Dose/Concentration	Potential targets		References
				Up	Down	
**Lung cancer**						
Cytotoxicity	PTK inhibition	NCI-H1435, NCI-H226, NCI-H460	30 μM		*HER*-2^ neu^	39
	Suppression of ERCC1 and Rad 51 *via* ERK_1/2_ inactivation	H1650, A549, H520, H1703	25-100 μM		ERCC1, Rad51, p-ERK_1/2_, MKK_1/2_	40
	Down-regulation of ERCC1 and Rad51	SK-MES-1, A549	40 μM, 70 μM		ERCC1, Rad51	41
	ERCC1 down-regulation and ERK_1/2_ inactivation	H520, H1703	8.1-24.3 μg/mL		ERCC1, p-ERK_1/2_	42
	Inhibition of ILK expression *via* increase of phosphorylation of AMPKα & ERK1/2 and suppression of Sp1 and c-Jun.	A549, PC9, H1299, H1650, H1975	50 μM	p-AMPKα	ILK, Sp1, c-Jun, p-ERK_1/2_	43
	Inhibition of cell growth and induction of cell cycle arrest at G2/M phase via activation of PPARγ & AMPKα/MEK/ERK, down-regulation of Sp1 and up-regulation of IGFBP1	A549, H1975 Nude mice (A549)	50 μM for cell; 25,50 mg/kg for mice	p-PPARγ, p-AMPKα, MEK, IGFBP1	Sp1, p-ERK_1/2_	44
	By inhibiting hyaluronan secretion and regulating the expression of cyclin, G1/G0 phase arrest was induced	A549, H520, H1975, H1299, H460	30 μM	Cyclin C, Cyclin D, Cyclin E	HAS2, Cyclin A, Cyclin B	45
Apoptosis	Emodin-induced cell death is closely associated with the mitochondria- dependent apoptosis	CH27	10, 50 μM	c-Caspase 3, c-Caspase 8, c-Caspase 9, Bak, Bax, Cyto C		46
	Induction of apoptosis *via* up-regulation of FASL and down-regulation of C-MYC	A549	16.85 μg/ml	FASL	c-Myc	47
	Induction of apoptosis *via* activation of ER stress and the TRIB3/NF-κB pathway	A549, H1299; BALB/c nu/nu nude mice (A549)	80 μM for cell; 50 mg/kg	c-Caspase 3, CHOP, TRIB3, GRP78		48
	Induction of mitochondria-dependent apoptosis via activating a ROS-elicited ATM-p53-Bax signaling pathway	A549	50 μM	p-ATM, p53, Bax, Cyct C	Survivin	15
	Induction of apoptosis *via* ROS generation and reduced ∆*Ψ*m	A549, H460, CH27, WI38	50 μM	c-Caspase 2, c-Caspase 3, c-Caspase 8, c-Caspase 9, Bax, ROS, Cyto C	Bcl-2, p-Akt, p-ERK_1/2_	49
	Induce tumor cell apoptosis	A549	9.31 μg/ml			50
	Inhibition of MTH1 promotes DNA damage and apoptosis of tumor cells	NCI-H-520, NCI-H-460, A549	25, 50, 75 μM	ROS, Cyclin B1, PARP, c-Caspase 3, Bax,	CDK4, Bcl-2, MTH1, CDK2, Cyclin D1, Survivin, VIM	52
	Inhibition of proliferation of non-small cell lung cancer *in vitro* and *in vivo*	A549, H1650, H460, H1975, PC9, H1299 C57 mice (LLC cells)	20, 40, 60 μM for cell; 25, 50 mg/kg	ROS, Bax, P27, p-AMPK	sPLA2-IIa, NF-κB P65, IKKβ, IκBα, p-mTOR, p-ACC, p-PKM2, p-AKT, Cyclin D1, Cyclin B1, Bcl2	53
	Increase ROS, reduce autophagy, induce lung cancer cell apoptosis *in vivo* and *in vitro*	LLC cell; ICR mice (urethane-induced lung carcinogenesis)	20 μM for cell; 10 mg/kg for mice	IFN-γ, IL-12, ROS, P62	IL-6, TNF-α, TGF-β1, LC3-B	60
Autophagy	Induction of autophagy *via* the mutation independent p53 aggregation.	A549	10, 15, 20 μM/70 μM	p53, LC3	ERCC1, Rad51	16,41
Inhibition of metastasis and invasion	Down-regulation of CXCR4 and HER2	A549	100 μM		CXCR4, *HER*-2^ neu^	54
	Inhibition of ATP-induced proliferation and migration by suppression of P2Y receptor and Ca^2+^ dependent NF-κB pathway	A549	1, 5 μM	Bax, Claudin-1, E-cadherin	Bcl-2, Fibronectin, SNAIL, NF-κB p65	55
	Suppressing expressions of Twist, SNAIL & Slug, and inhibiting activation of NF-κB	H69, H69AR	10,20,50 μM		Twist, SNAIL, Slug, NF-κB p65	56
Chemotherapy resistance	Inhibition of drug efflux enhances cisplatin-induced apoptosis and DNA damage	A549, H460	2.5, 5, 10 μM		Pgp	57
	It synergistically inhibited the proliferation of A549 cells with paclitaxel *in vivo* and *in vitro*, and exerted anti-tumor effect	A549; BALB/nude mice (A549)	10 μM for cell; 50 mg/kg for mice	Bax, c-Caspase 3	Bcl-2, p-Akt, p-ERK_1/2_	58
	Reverse cisplatin resistance, promote lung cancer cell apoptosis, inhibit cell migration and invasion	A549	Not mentioned		NF-κB, P-gp, MDR-1, GST	59
**Hepatocellular carcinoma**						
Cycle arrest	G2/M phase arrest of tumor cells	Huh7, Hep3B, HepG2	50 μM	Cyclin B, Chk2, Cdk2, P27, CYP1A1, CYP1B1, CHAC1, TIPARP, GDF15, SOS1, RASD1, SLC7A11, CYR61, MRAS, SERPINE1	Cdc25c, P21, NR1H4, PALMD, TXNIP, IGFBP3, Cyclin A, Cdk1	65
	Results in G1 phase arrest, increased intracellular ROS level and DNA fragmentation	HepG2	30, 60, 90, 120 μM	c-Caspase 8, c-Caspase 9, Cyto C, p53	Bcl-2, NF-kB p65, p-Caspase 3	66
	It can cause G1 phase arrest and cytotoxicity, increase ROS level and inhibit cell glycolysis	HepG2	10, 20, 40 μM		PKM2, HK11, LDHA	67
Apoptosis	ROS production is increased, G2/M phase arrest occurs, and the mitochondrial transmembrane potential (∆*Ψ*m) decreases, leading to DNA fragmentation and inducing cell apoptosis	Mahlavu, PLC/PRF/5, HepG2	Mahlavu (5, 10, 30 μg/ml), PLC/PRF/5 (40, 80, 160 μM), HepG2 (20, 40, 80 μM)	Cyt c, P53, P21, Bax, Cyclin E, c-Caspase 3, c-Caspase 9, c-PARP	Bcl2, Cyclin A, CDK2	68-70
	Decreased mitochondrial membrane potential (∆*Ψ*m) and induced apoptosis	HepG2	50, 100 μM	CypD, Cyt c	p-ERK_1/2_	71
	Induce tumor cell apoptosis and inhibit tumor growth	HepG2, PLC/PRF/5, Hep3B, C3A; Athymic nu/nu female mice (HCCLM3)	10, 50 μM for cell; 25, 50 mg/kg for mice	SHP-1, c-Caspase 3; PARP	CD31, p-STAT3, Bcl2, Bcl-xL, survivin, Mcl-1, VEGF, p-JAK2, p-JAK1, p-AKT, p-Src, cyclin D1	76
	Inhibit cell viability and promote tumor cell apoptosis through death receptor and mitochondrial pathways	HepG2, HL-7702	20, 40, 80 μM	PARP, BAX, Cyt c, Fas, Fas-L, tBid, p-p38	p-Caspase 3, Bcl2, Bid, p-Caspase 8, p-Akt, p-ERK_1/2_, p-JNK	77
	Decrease cell viability and induce apoptosis *in vitro*. Inhibit tumor growth *in vivo*, induce apoptosis of tumor cells, improve liver and kidney function of tumor mice	SMMC-7721 male BALB/c-nu nude mice (SMMC-7721)	25, 50, 100 μM for cell; 25, 50 mg/kg for mice	p-p38, c-Caspase 3, c-Caspase 9	p-AKT, p-Caspase 9, p-JNK, p-ERK1/2, p-Caspase 3	78
	Induce tumor cell apoptosis and inhibit tumor growth	HepG2; BALB/c nude mice (HepG2)	10, 100 nM for cell; 1, 10 mg/kg for mice	mir-34a	SMAD2, SMAD4, p-VEGFR2, p-AKT, p-ERK_1/2_	79
	Inhibit lipid metabolism of tumor cells, promote apoptosis and inhibit tumor growth	BALB/C mice (Be L-7402)	20, 40, 80 mg/kg	Bax, c-Caspase 9, c-Ccaspase 3, APAF1, Cyt c, AIF	Bcl2, SREBP1, FASN, ACACA, ACLY, SCD1, SIP, SCAP、Caspase 2	80
	It inhibited tumor cell viability, reduced mitochondrial membrane potential, inhibited triglyceride level and fatty acid desaturation, and induced apoptosis	Bel-7402	100 μM	c-Caspase 3, c-Caspase 9, APAF1, Cyt c, ENDOG, AIF, Bax	Bcl2, SCD, FASN, ACACA, ACLY, SREBP1	81
Inhibition of metastasis and invasion	Inhibit the migration and invasion of tumor cells and inhibit lung metastasis *in vivo*	HepG2, Hep3B, PLC/PRF5, HUH7 female Balb/c nude mouse (HCCLM3)	50 μM for cell; 25, 50 mg/kg for mice		CXCR4, HER2, NF-kB	84
	Inhibit tumor cell viability, induce a small amount of apoptosis, inhibit cell migration and invasion	MHCC-97H	100 μg/kg	p-p38	p-ERK_1/2_, p-Akt, MMP-2, MMP-9	85
Chemotherapy resistance	Reversal of cisplatin resistance increases DNA damage	HepG2	10 μM		FGFR2, p-ERK_1/2_, ERCC1	89
	Enhanced irradiation induces cytotoxicity G2/M block was induced and apoptosis was induced	HepG2	10 μM	c-PARP1	JMJD1A, HIF-1α, JMJD2B	90
	It can induce G1 phase arrest and apoptosis, reduce cholesterol synthesis, inhibit tumor growth, and improve Sorafenib resistance	HepG2, Hep3B, Huh7, SK-HEP-1, PLC/PRF5; BALB/c-nude mice (HepG2 or SK-HEP-1)	20 μM for cell; 10 mg/kg for mice	c-Caspase 3	HMGCS1, HMGCR, FDPS, p-AKT, p-4E-BP1, p-STAT3	91
	Enhance the toxicity of cisplatin and inhibit the migration and invasion of tumor cells	HepG2 cell	25, 50 μg/ml	E-cadherin		92
**Colon cancer**						
Cell cycle	Intracellular ROS production and Ca^2+^ release were induced, and G0/G1 phase arrest was induced in tumor cells	LS1034; Athymic BALB/c nu/nu mice (LS1034)	10, 20, 30, 40, 50 μM for cell; 40 mg/kg for mice	c-Caspase 3, c-Caspase 9, Bax, AIF, Cyt c	Bcl2	96
Apoptosis	Increase intracellular ROS production and induce tumor cell apoptosis	HCT116	20, 40, 80 μM	Bax, Cyt c, P53	Bcl2	97
	Inducing tumor cell apoptosis through mitochondrial pathway	LOVO	10, 20, 40 μM	Bax, Cyt c,	Bcl2	98
	Increase intracellular ROS level, inhibit tumor cell proliferation and induce apoptosis.	SW480, SW620	20, 40, 60, 80 μM	p-P38, P53, Puma		99
	Inhibition of fatty acid synthesis of tumor cells plays an anti-proliferation and pro-apoptotic role	HCT116, SW480	25 μM		FASN, p-AKT, p-PI3K	100
	Regulation of PI3K/AKT pathway induces G2/M cycle arrest and apoptosis of human colon cancer cells	CACO-2	15, 30, 60 μM	Bax	Bcl2, p-PI3K, p-Akt	101
	Induce cell apoptosis, inhibit migration and invasion, inhibit tumor growth, and reverse 5-FU resistance	SW480, SW480/5-Fu BALB/c nude mice (SW480/5-Fu)	9 μM for cell; 40 mg/kg for mice	Bax, c-Caspase 3	Bcl2, p-ERK_1/2_, p-AKT	102
Autophagy	Increase intracellular ROS accumulation, induce cell apoptosis and autophagy	HCT116, LOVO	20 μM	c-Caspase 9, c-Caspase 3, c-PARP, LC3-2, Beclin 1, LC3-1, Cyt c, Bax	P62, Bcl2	103
Inhibition of metastasis and invasion	Inhibit the migration and invasion of tumor cells	DLD-1	10, 20, 30, 40 μM	α- ERM pThr567	PRL-3	104
	The ROS level in tumor cells was increased, G2/M phase arrest occurred, and the migration and invasion of tumor cells were inhibited	SW480, SW620	50 μM	CDH1, EP300	β-catenin, TCF, LEF, hbp1, PCNA, Cyclin D1, c-Myc, SNAIL, VIM, MMP-2, MMP-9	105
	Blocking EMT, and inhibits the invasion and migration of tumor cells *in vivo* and *in vitro*	HT29, RKO Balb.c nude mice (RKO)	5, 10, 20 μM for cell; 40 mg/kg for mice	E-cadherin	VEGF, MMP-7, MMP-9, N-cadherin, SNAIL, N-catenin, TCF4, Cyclin D1, c-Myc	106
	Inhibit the growth, adhesion and migration of HCT116 cells, and inhibit the growth of xenograft tumor	HCT116; BALB/c nude mice (HCT116 cells)	15, 30, 60 μg/ml for cell; 20, 40, 80 mg/kg for mice		VEGFR2, p-PI3K, p-AKT	107
Anti-inflammatory	Inhibit intestinal inflammation related to cancer and prevent the occurrence and progression of intestinal tumors	SW620, HCT116 AOM/DSS model mice	10, 20 ,40 μM for cell; 50 mg/kg for mice		TNFa, IL1a/b, IL6, CCL2, CXCL5, COX-2, iNOS	108
**Breast cancer**						
Cytotoxicity	Inhibit the growth of cancer cells, induce the production of lipid droplets, and promote the mature differentiation of BC cells	MDA-MB453, BT-483, MDA-MB231, MCF-7	40 μM		HER-2/*neu*	111
Apoptosis	Apoptosis is induced by mitochondrial signaling pathway	BCap-37	20, 50 μM	Bax, Cyt-c	Bcl2	115
	Apoptosis is induced by the destruction of mitochondrial signaling pathways in cells	BCap-37	20, 50 μM	P21, P53	IGF-2	116
	Induce DNA breakage and DNA fragmentation, and induce tumor cell apoptosis and cycle arrest through internal and external pathways	MCF-7	30 μg/ml	Fasl	Mcl-1, Cyclin D, c-MYC	117
	Inhibition of ERα pathway and PI3K/Akt pathway inhibited the proliferation of tumor cells and induced apoptosis	MCF-7, MDA-MB-231	20, 40 μM		ERα, Cyclin D1, BCL2, p-MAPK, p-AKT	118
	Induce growth inhibition and apoptosis of human breast cancer cells	Bcap-37, ZR-75-30	10, 40 μM	c-Caspase 3, PARP, p53, Bax	Bcl-2	119
	It exerts anti-tumor activity by activating AhR-CYP1A1 signaling pathway	MCF-7	25, 50, 100 μM	AHR, CYP1A1		120
Chemotherapy resistance	Increase tumor sensitivity to paclitaxel and improve tumor drug resistance	MDA-MB-361, MDA-MB-453, BT-483, SKBr-, BT474, MDA-MB-231, MCF-7; Nu/nu mice (MDA-MB-361 or MDA-MB-231)	20 μM for cells; 40 mg/kg for mice		HER-2/neu	113
	Inhibit DNA damage repair and reverse multidrug resistance of tumor cells	MCF-7/Adr MCF-7	20 μg/ml		ERCC1	123
	Enhance apoptosis of breast cancer cells, resulting in cell senescence	MCF-7	20 μM	P21, P16, P27, ROS	E2F1, NRARP, GSH	124
	It increased the sensitivity of BC cells to doxorubicin, inhibited cell proliferation and induced DNA damage	MDA-MB-231, MCF-7	110 μM	γH2A, P53	AKT1, XRCC1, PARP1, RAD51	125
Inhibition of metastasis and invasion	Inhibition of tumor cell metastasis by targeting HER-2/ neu	MDA-MB453, MCF-7	20 μM		HER-2/neu	112
	Inhibits the invasion of breast cancer cells *in vivo* and *in vitro*	MDA-MB-435s, MDA-MB-468	1, 10 μM		P2X7R	126
	It can reduce the infiltration of macrophages, reduce the migration of macrophages to tumor environment, inhibit the polarization of macrophages M2, and inhibit the lung metastasis of tumor	4T1 cell, EO771 BALB/c or C57BL/6 mice (4T1 cell and EO771 cell)	10, 100 μM fo cells; 40 mg/kg for mice		p-STAT6, C/EBPβ	129
	Inhibit the EMT of breast cancer cells and the formation of cancer stem cells, and prevent the recurrence of lung metastasis after breast cancer	EO771, 4T1, MCF7, MDA-MB-231 C57BL/6, BALB/c, NOD-SCID mice (EO771, 4T1, MCF7, MDA-MB-231)	40 mg/kg		TGF-β1	130
	Inhibit macrophage infiltration and m2-like polarization, block their migration and adhesion to the tumor site, inhibit tumor growth, increase T cell activation, and reduce tumor angiogenesis	4T1, EO771 C57BL/6 and BALB/c mice (4T1 cells, EO771 cells)	0-100 μM for cells; 40 mg/kg for mice	iNOS	MMP 2, MMP 9, JMJD3, Arg1, p-STAT6, C/EBPβ, CSF-1, MCP-1, ICAM1, Thy1	131
	Inhibit TGF-β and inhibit the EMT and migration of cancer-associated fibroblasts	BT20	30 μM	E-cadherin	β-catenin, VIM, MMP-2	132
	Inhibit tumor cell migration *in vivo* and *in vitro*, and inhibit lung metastasis of breast cancer in nude mice	MDA-MB-231 athymic nude mice (MDA-MB-231)	10, 20, 40, 80 μM for cells; 40 mg/kg for mice		MMP 2, MMP 9, uPA, uPAR, p38, p-ERK_1/2_	133
	Inhibit CCL5 secretion of adipocytes, inhibit EMT of tumor cells, inhibit tumor growth and lung liver metastasis	MDA-MB-231, MDA-MB-453 Balb/C nude mice (MDA-MB-231)	50 μM for cells; 40 mg/kg for mice	GSK3, E-cadherin	CCL5, p-AKT, β-catenin, vimentin, SNAIL, p-CCR5, MMP2, MMP9	134
Anti-angiogenesis	Tumor cell - induced metastasis and angiogenesis were inhibited *in vitro* and *in vivo*	EA.hy 926; NOD/SCID mice/ SD rats (MDA-MB-231)	10, 20, 40 μM for cells; 40, 80 mg/kg for mice		MMP9, MMP13, p-Runx2, p-VEGFR-2	135
	Inhibit angiogenesis and tumor growth	MDA-MB-231, 4T1; BALB/c NOD-SCID mice; and, BALB/c mice	5, 10, 20 μM for cells; 10 mg/kg	SerRS, HOXB1, PCK1, UCP1, NCOR2, HDAC3	VEGFA	136
**Pancreatic cancer**						
Cytotoxicity	Promote the demethylation of tumor suppressor genes and inhibit the growth of pancreatic cancer cells	PANC-1	10,20,40μM	P16, RASSF1A, ppENK	5mC, DNMT1, DNMT3a	145, 146
	Inhibit tumor cell growth, angiogenesis and glycolysis, reduce cancer cachexia	AsPC-1, BxPC-3, HPAF-2, MiaPaCa2, Panc-1; Male athymic Balb/c mice (MiaPaCa2)	100 μM for cells; 50 mg/kg for mice		HIF-1α, Glut1, HK-II, PFK- 1, VEGF, caveolin-1, p-Akt, p-ERK_1/2_, PHD-2	148
Apoptosis	It plays anti-tumor proliferative role by inducing apoptosis	Mia Paca-2, BxPC-3, panc -1, L3.6pl	12.5, 25, 50 μM	PARP		149
	Induced apoptosis of pancreatic cancer cells and increased sensitivity of pancreatic cancer to gilotrif	PANC-1, BxPC-3 BALB/c nude mice (PANC-1)	30, 60, 90 μM for cells; 50 mg/kg for mice	c-Caspase 3, bax	p-STAT3, Bcl2, EGFR	153
Chemotherapy resistance	Enhanced the antitumor activity of gemcitabine	Mia Paca-2, BxPC-3, panc -1, L3.6pl	40, 80 μM	c-Caspase 3, PPAR	Survivin, b-catenin	154
	Enhanced the antitumor activity of gemcitabine	SW1990, SW1990/GZ	20 μM		NF-κB	155
	Increased sensitivity of tumor cells to gemcitabine	SW1990; BALB/c female mice (SW1990)	40 μM for cells; 40 mg/kg for mice	Bax, CytC, c-Caspase 3	Bcl-2	156
	Improve chemotherapy resistance of tumor cells to Gemcitabine	BALB/c female mice (SW1990)	40 mg/kg for mice	Bax, c-Caspase 9, c-Caspase 3, CytC	p-AKT, Bcl-2, NF-κB p65	157
	Enhanced the antitumor activity of gemcitabine	SW1990; Female BALB/c nude mice (SW1990)	40 μM for cells; 40 mg/kg for mice		XIAP, NF-Κb p65	158
	Enhanced the antitumor activity of gemcitabine	BaLB/c male mice (Panc-1)	40 mg/kg	c-Caspase 9, c-Caspase 3	XIAP, NF-κb p65, Survivin	159
	Increased sensitivity of tumor cells to gemcitabine	SW1990, SW1990/GZ	10, 20, 40, 80, 160 μM	Bax, Cytc, c-Caspase 9, c-Caspase 3	MDR-1 (P-gp), NF-κB p65, Bcl-2	160
	Increased sensitivity of resistant cells to gemcitabine treatment	Bxpc-3/Gem	40 μM		MDR-1 (P-gp), NF-κB p65, XIAP, survivin	161
	To enhance the therapeutic effect of gemcitabine and improve the drug resistance of tumor cells to gemcitabine	BALB/c mice (PANC‑1)	40 mg/kg		MDR-1(P-gp), MRP1, MRP5	162
	Reversal of gemcitabine resistance in pancreatic cancer cell lines	pan -1/Gem, MIAPaCa-2/Gem	40 μM	c-Caspase 3, c-Caspase 9, IκB-α	Survivin, XIAP, NF-κB p65, IKKβ, P-gp	163
Inhibition of metastasis and invasion	Inhibit metastasis of pancreatic cancer	SW1990; BALB/c nu/nu mice (SW1990)	10, 20, 40 μM for cells; 20, 40 mg/kg for mice	c-Caspase-3	MMP9, NF-κB p65, survivin	164
	Inhibit EMT and invasion of pancreatic cancer cells, and inhibit hepatic metastasis of pancreatic cancer	SW1990; Nude mice (SW1990)	20, 40 μM for cells; 50 mg/kg for mice	miR-1271, E-cadherin	ZEB1, TWIST1	166
Anti-angiogenesis	Regulating the expression of angiogenesis related factors can promote apoptosis and inhibit angiogenesis	SW1990, Panc-1, ECs Female athymic BALB/c nu/nu mice (Panc-1 cells)	40 μM for cells; 1 mg/mouse for mice		NF-κB p65, VEGF, MMP 2, MMP 9, p-eNOS	167
	Inhibits angiogenesis in pancreatic cancer	SW1990; Female athymic BALB/c nu/nu mice	20, 40, 80 mg/kg for mice	miR-20b, Smad4, TβRI, TβRII	TGF-β1, Angptl 4, miR-155, miR-210	168
**Leukemia**						
Cytotoxicity	Induction of ROS production, improve the sensitivity of tumor cells to arsenic trioxide	C8166 cells, MT2, II85, LAF, Jurkat	10 μM	PARP, ROS	Akt, Jun D, JAB 1	171
Apoptosis	Apoptosis of HL-60 cells was induced by ROS independent method	HL-60	40 μM	Caspase3, PARP, D4-GD1,	Mcl-1,	172
	G0/G1 phase arrest was induced and apoptosis was induced	K562	20, 40, 80, 100 μM		c-myc	173
	Apoptosis of tumor cells was induced by caspase signaling pathway	K562	20, 30, 40 μM	c-Caspase 3, c-Caspase 9, c-Caspase 8		174
	Inhibit the growth of tumor cells *in vivo* and *in vitro* and induce their apoptosis	BALB/c nude mice (K562)	25, 50, 100 mg/kg	Bax	Bcl2	175
	Cause tumor regression and induce cell apoptosis	K562; Male BALB/c nude mice (K562)	25, 50, 100 μM for cells; 20, 50 mg/kg for mice	Bax, c-Caspase 3, c-Caspase 8, c-Caspase 9	Bcl2	176
	Induce G0/G1 phase arrest and apoptosis	U937	30, 60, 90 μM	Bax	Bcl2, CPP32	177
	Apoptosis of human myeloma cells was significantly induced by inhibition of McL-1	RPMI8226, U266, IM-9	10, 20, 50 μM	c-Caspase 3, c-Caspase 9	p-JAK2, p-STAT3, Mcl-1, Histone H2	178
	Inhibit hL-60 cell proliferation, induce G0/G1 phase arrest, and induce apoptosis	HL-60	10, 20, 40 μM		p-AKT, p-IκB-α, p-p65, p-mTOR	179
	Decreased cell mitochondrial membrane potential, caused cell G0/G1 phase arrest, induced apoptosis, improved doxorubicin resistance	HL-60 (ADR)	10, 20, 40 μM	c-Caspase-3	Bcl-2, c-myc、	180
	Induction of tumor cell apoptosis, overcoming all-trans retinoic acid resistance	NB4, MR2, primary AML	10, 30, 60 μM	c-Caspase 9, c-caspase 3, PARP	Bcl-2, RARα, p-Akt, p-mTOR, 4E-BP1, p70S6K	181
	Inducing apoptosis of tumor cells *in vivo* and *in vitro*	K562; BALB/c nude mice (K562)	25, 50, 100 μM for cells; 25, 50, 100, 120 mg/kg for mice	PTEN	PI3K, AKT, BCR-ABL	182
	Decreased cell viability, induced DNA damage, decreased ΔΨm levels, and induced apoptosis through endoplasmic reticulum stress (ER) and mitochondrial pathways	WEHI-3;Male BALB/c mice (WEHI-3)	25, 50, 100 μM for cells; 5, 10 mg/kg for mice	ROS, c-Caspase 8, c-Caspase 9, Cyt-c, c-Caspase 7, c-Caspase 12, c-Caspase 3, PARP, Apaf-1, AIF, Endo G, GADD153, GRP78, ATF-6α, Bax, Bad	Bcl2, Bcl-xl	183
Chemotherapy resistance	Increase the sensitivity of resistant cells to chemotherapeutic drugs	K562/ADM	6.1, 17.6, 33.2 μM		MDR1, P-gp	187
	The doxorubicin resistance of K562/ADM cells was reversed	K562/ADM	50, 100, 200 μM		P-gp	188
	Increased cytotoxicity of 3'-azido-3'-deoxythymidine to tumor cells	K562	8, 16, 32 μM	EGR1	β-catenin	189
	The chemosensitivity of AML cells to ARA-C was increased, and the survival rate of AML transplanted tumor mice was improved.	HL-60/ADR; BALB/C-nude mice (HL-60/H3)	5, 10 μM for cells; 20, 40 mg/kg for mice	PARP, c-Caspase 9, c-Caspase 3, Bax	Bid, p-Akt, p-mTOR, p-4E-BP1, p-ERK_1/2_, p-P70S6K, Bcl2	190
	Enhanced the sensitivity of drug-resistant cells to imatinib, inhibited cell proliferation and induced cell apoptosis	K562, G01	20, 40 μM	c-Caspase-3, c-PARP	p-Bcr-Abl, c-MYC, MCL-1, Bcl-2, p-STAT5, Src, p-Src	191
**Cervical cancer**						
Cytotoxicity	It inhibited the proliferation of HeLa cells and reduced the tumor growth of tumor-bearing mice	HeLa; Female old athymic nude mice (HeLa)	1, 10, 25 μM for cells; 25 mg/kg for mice	p-STAT1, p-STAT2, IFNAR1, p-TYK2	p-STAT3	193
Apoptosis	Inhibits DNA synthesis and induces apoptosis through the mitochondrial pathway	HeLa, Ca Ski, ME-180, Bu 25TK	25, 50 μM	c-Caspase 3, c-Caspase 9		194
	Induce tumor cell apoptosis	HeLa	40 μM	p-JUN	p-AKT, mTPR, p-PTEN, P-MAPK	195
	Apoptosis is induced by internal mitochondrial and external death receptor pathways	HeLa	20, 40, 80 μM	caspase-3, caspase-9, caspase-8, Fas, Fasl, FADD, Cyt-c, Apaf-1	JAK2, STAT3, Mcl-1	196
	Induce apoptosis and autophagy, inhibit cell cycle, inhibit angiogenesis	Hela, JAR, HO-8910	5, 10, 15 μM	Atg12-Atg5, Beclin-1, c-Caspase-9, c-Caspase-3	Cyclin D1, Cyclin E1, VEGF, VEGFR-2, Bcl2, Mcl-1, MAPLC3	197
Autophagy	By increasing the number of lysosome, the number of autophagic vacuoles and the activity of lysosome hydrolase can induce lysosome membrane damage and promote the death of tumor cells	HeLa	1, 15, 30, 60, 100 μM	Cathepsin D, Cathepsin L		198
	To improve the toxicity of photodynamic therapy to cervical cancer cells and increase the activity of caspase-3 and autophagy	SiHa, CaSki	30 μM	c-Caspase 2, ROS, ATF2, AURKA, AURKC, BIRC5, CDK1, CDK7, GSTP1, HDAC4, HIF1A, HSP90AA1, MDM4, MTOR, PARP4, PIK3C2A, PIK3C3, PIK3CA, PLK2, PLK4, RHOA, RHOB, TNKS, TOP2B	CTSS, ESR1	199
Inhibition of metastasis and invasion	Inhibit the invasion, migration and stem cell characteristics of tumor cells and reverse EMT	SiHa, Hela	20 μM	Bax	TGFRII, Smad2, Smad3, Smad4, CyclinD1, p21, Pin1, p15, p16, CDK6, p27, SNAIL, Slug, Bcl 2, β-catenin	200
**Ovarian cancer**						
Cytotoxicity	Induced DNA damage and inhibited cell proliferation	A2780	1 μM			202
	Inhibit cell viability, and reduce cell viability and colony formation of A2780 cells	A2780	20 μM	FOXD3, miR-199a	TGF-β2	203
Apoptosis	Inhibit tumor cell proliferation, induce apoptosis and inhibit invasion	SKOV3, HO8910	20, 60 μM		surviving	204
Inhibition of metastasis and invasion	Inhibit EMT, migration and invasion of tumor cells, and inhibit metastasis of ovarian cancer	A2780, SK-OV-3	20, 40, 80 μM	E-cadherin, Claudin	N-cadherin, vimentin, p-GSK-3β, ILK, β-Catenin, and Slug	205
	Inhibit EMT and invasion of tumor cells	A2780, SK-OV-3	10, 20, 40 μM	E-cadherin, keratin	N-cadherin, Vimentfin, MMP 9, MMP 2, ZEB1, p-GSK-3β, β-Catenin	206
	Inhibit the proliferation, migration and invasion of ovarian cancer cells	A2780, SK-OV-3; Female BALB/C nude mice (SK-OV-3)	20 μM for cells; 50 mg/kg for mice	E-cadherin	Slug, MMP 9, Vimentin, ILK	207
Chemotherapy resistance	Induced apoptosis and increased sensitivity of drug-resistant cells to paclitaxel	A2780	10 μM	c-Caspase 3	P-gp, XIAP, MDR-1, surviving	208
	Increase the sensitivity of drug-resistant cancer cells to cisplatin	COC1	50 μM	ROS	MRP1	209
	Inhibit the growth of cancer cells and enhance the sensitivity of drug-resistant cells to cisplatin therapy	SKOV3, OVCAR3, MDH2774, and ES2	0-50 μM		AURKA	220
**Head and neck neoplasm**						
Cytotoxicity	Induced cell DNA damage	SCC-4	25, 50, 100 μM		ATM, ATR, 14-3-3σ, BRCA1, DNA-PK, MGMT	212
	Inhibit the growth, proliferation and cell division cycle of human oral squamous cell carcinoma cells	Tca8113	10, 20, 40, 80 μM		CDK2, Cyclin E, P21	216
	Inhibition of cell cycle markers play an anti-proliferation role	Buccal mucosa of hamsters treated with DMBA	50 mg/kg		Cyclin D1, PCNA, CDK4, CDK6, survivin	217
	Prevention of DMBA - induced hamster buccal pouch carcinogenesis by proapoptotic and antioxidant effects	Buccal mucosa of hamsters treated with DMBA	50 mg/kg	P53, Bid, Bax, c-Caspase 3, c-Caspase 9	Bcl-xl	218
Apoptosis	Increased ROS level leads to DNA damage, endoplasmic reticulum stress and apoptosis of tumor cells	SCC-4	30 μM	ROS, c-Caspase 9, c-Caspase 3, P21, Chk2, Cyto c, AIF, GADD153, GRP78, Bax	Cyclin B1, Cdc2, Bcl2	211
	Apoptosis was induced by the production of ROS and the decrease of pH	EC-109	2.5, 5, 10, 20 μM	ROS	Intracellular PH	214
	Tumor cell death was induced by apoptosis and necrosis	HSC-3	46.3, 92.5, 185 μM	Bax, c-Caspase-9, c-Caspase-3	Bcl2, p-AKT	219
	Induce cell cycle arrest and apoptosis	Human nasopharyngeal carcinoma cells (CNE-2Z)	50 μM	chloride channel		220
	Inhibit the proliferation of thyroid papillary carcinoma cells, induce cell cycle arrest and apoptosis	TPC‑1; Balb/c female nude mice (TPC‑1 cells)	10, 25, 50 μM for cells; 40 mg/kg for mice	p-AMPK, c-Caspase 3, Cyclin D1	PCNA, p-MEK, p-ERK_1/2_	222
Inhibition of metastasis and invasion	Inhibit tumor cell migration and invasion	SCC-4	15,30μM	TIMP-1	MMP 2, u-PA, FAK, NF-κB p65, p-AKT, p-P38, p-JNK, p-ERK_1/2_	213
	Inhibit EMT of tumor cells and inhibit migration and invasion	FaDu, HEK-293T, OECM-1; Severe combined immunodeficient (SCID) mice	5 μM for cells; 50 mg/kg for mice	E-cadherin, p-GSK-3β	TEIST1, Vimentin, p-AKT	215
	Inhibit tumor angiogenesis and lung metastasis	8505c, SW1736 Balb/c nude mice (SW1736)	10, 15, 20, 25 μM for cells; 100 mg/kg for mice		TRAF6, HIF1α, VEGF, CD147, MMP 9	221
**Glioma**						
Inhibition of metastasis and invasion	The invasion of hyaluronic acid (HA) induced glioma cells was inhibited	Hyaluronic acid (HA)-induced invasion of human glioma cells.	40 μM		MMP2, MMP9, p-FAK, p-ERK_1/2_, p-Akt, p-PKB, AP-1, NF-kB-p65	223
	Inhibit cell migration and intracellular glycolysis	U-87 MG or ΔFBP1 U-87 MG male BALB/c athymic nude mice (U-87 MG or ΔFBP1 U-87 MG)	20, 40 μM for cells; 40 mg/kg for mice	E-cadherin	FBP1, Vimentin, Fibronectin	224
Apoptosis	Apoptosis of glioma stem cells was induced, the invasiveness of glioma stem cells was decreased, and the sensitivity of glioma stem cells to ionizing radiation was increased	X01 and X03, and CSC2	5 μM	Hsp90	b-catenin, p-STAT3, p-Akt, SNAIL, slug, p-EGFR	225
	The proliferation of U251 cells was inhibited and apoptosis and necrosis were induced	U251; BALB/C nude mice (U251)	10, 20, 40 μM for cells; 20, 40, 80 mg/kg for mice	TNF-α, RIP 1, RIP 3, MLKL, c-Caspase-3	Caspase 8	226
**Neuroblastoma**	Inhibit the migration and invasion of tumor cells *in vitro*	SH-SY5Y	10, 25 μM		GRB2, RhoA, NF-kB p65, HIF-1a, VEGF, FAK, Ras, COX2, p-p38, p-JNK, MMP2, MMP9, MMP7	227
	Trigger caspase cascade signaling pathway and induce tumor cell apoptosis	IMR-32	20 μM	Ca^2+^, ROS, p53, p21, c-Caspase-9, c-Caspase-3		228
**Prostatic cancer**	Inhibit the growth of tumor cells and prolong the survival time of tumor mice	LNCaP, PC3, DU-145 Male athymic nude mice (PC3)	10, 20, 40 μM for cells; 40 mg/kg for mice	PARP	AR, PSA	229
	Inhibit cell proliferation and induce cell apoptosis through mitochondrial pathway	LNCaP, PC-3	10, 20, 30, 40 μM	p53, p21, Bax, c-Caspase 3, c-Caspase 9	Bcl-2, AR, PSA	230
	Enhance anti-tumor effect of cisplatin *in vivo* and *in vitro* and reverse drug resistance	DU-145; BALB/c-nu/nu mice (DU0145)	50 μM for cells; 50 mg/kg for mice	ROS	MDR1, HIF-1	231
	Inhibit tumor growth	LNCaP, PC-3	50 μM	ROS, LRP1	AR	232
	Inhibit the migration and invasion of tumor cells	DU145	100 μM		CXCR4, HER2, NF-Κb p65	54
	Inhibit the growth of tumor cells and induce cell cycle arrest and apoptosis	PC-3	10, 20, 40, 60, 80 μM	Notch1	Jagged1, VEGF, bFGF	233
**Bladder cancer**	Reverse the transformation of cancer epigenetics to normal epigenetics, and inhibit the occurrence of tumors	T24, TSGH8301, MBT24	40, 80 μM	H3K27me3	HBP17, FABP4, pH3Ser10	234
	Improve cisplatin resistance of tumor cells *in vitro* and *in vivo*	T24, J82; BALB/cnu/nu mice (T24)	20 μM for cells; 50 mg/kg for mice	ROS	MRP1	235
**Lymphoma**	Mitochondrial apoptosis pathway of Dalton's lymphoma (DL) cells was induced *in vivo*	Inbred AKR strain mice (DL cells)	40 mg/kg	H_2_O_2_, Bax, Cyto c, SOD2, SOD1	Bcl2, GPx	236
	It can reduce the survival rate of tumor cells, induce apoptosis and increase the sensitivity of tumor cells to doxorubicin	Raji	6.25, 12.5, 25, 50 mg/kg	c-Caspase 3, c-Caspase 9, PARP, DNMT3A	p53, UHRF1	237
	Inhibit cell proliferation and induce cell apoptosis	SU-DHL4	10, 20, 40 μM		p-PI3K, P53, p-AKT	238
**Gallbladder carcinoma**	Induce apoptosis and improve the sensitivity of tumor cells to chemotherapy	SGC996; BALB/c-nu/nu mice (SGC996)	50 μM for cells; 50 mg/kg for mice	ROS	GSH, MRP1	241
	Cisplatin-induced apoptosis of gallbladder carcinoma cells is promoted in a ROS dependent manner	SGC996; BALB/c-nu/nu mice (SGC996)	50 μM for cells; 50 mg/kg for mice	ROS	surviving	242
**Osteosarcoma**	*In vitro* and *in vivo* anti-angiogenesis of osteosarcoma	SOSP-9607, MG63, SAOS-2; Male BALB/c nude mice (SOSP-9607, MG63, SAOS-)	2.5 μM for cells; 0.3 mg/kg for mice	SIRT1	VEGF, H4-K16AC	243
	Reduce the radiation resistance of tumor cells and promote cell apoptosis	MG63	15, 30, 45, 60 μM	c-Caspase 3	Shh, bcl2, Gli 1	244
	Apoptosis is induced by mitochondrial pathway and endoplasmic reticulum stress	U2OS	120 μM	ROS, GRP78, CHOP, c-Caspase 4		245
	Inhibit tumor cell proliferation and synergistic anti-tumor with cisplatin	MG-63	10 μM	Nrf2		246
**Skin cancer**	Inhibit skin tumor formation in mice	ICR mice skin tumors induced by 7,12-dimethylbenz[a]anthracene as an initiator and 12- O-tetradecanoylphorbol-13-acetate (TPA)	/			247
	It induced the disorder of cell redox balance and accelerated cell apoptosis	B16F10; C57BL6J mice (B16F10)	30 μM for cells; 5 mg/kg for mice	8-OH-dG, MDA, ROS, c-PARP, Drp1, Bax,	IDH2, p-4EBP1, p-P38, p-ERK_1/2_, OPA1	248
	Inhibit the proliferation and migration of tumor cells and induce G2/M phase cycle arrest	B16-F10; C57BL/6 (B16-F10)	20, 50 μM for cells; 50 mg/kg for mice		CD155	249
	Inhibit glycolysis of tumor cells and inhibit their proliferation	B16F10	4, 8 μM	p-AMPK	P53, AMPKα, ATP	250
	Inhibit the growth, migration and invasion of melanoma cells	B16F10, A375	20, 40, 60 μM	Bax	β-catenin, c-Myc, TCF, Bcl2, MMP2, MMP9	251
**Gastric carcinoma**	Induction of anoikis, a detachment-initiated apoptosis, in tumor cells, and increases the antitumor effect of arsenic trioxide	SGC-7901	5 μM	ROS, c-Caspase 3	RhoA	252
	The proliferation of SGC-7901 cells was inhibited and apoptosis was induced	SGC-7901	15, 30, 45, 60 μM		PRL-3	253

## Abbreviations

14-3-3σ: 14-3-3sigma
AhR: aromatic hydrocarbons receptor
APL: acute promyelocytic leukemia
AR: androgen receptor
ATC: anaplastic thyroid cancer
ATM: ataxia telangiectasia mutated
ATO: arsenic trioxide
ATP: adenosine 5ʹ-triphosphate
ATR: ataxia-telangiectasia and Rad3-related
AZT: 3'-azido-3'-deoxythymidine
BC: breast cancer
BRCA1: breast cancer 1 early onset
CML: chronic myeloGenous leukemia
CSC: cancer stem cell
CyPD: cyclophilin D
CRC: colorectal cancer
DL: dalton's lymphoma
DNA-PK: DNA-dependent serine/threonine protein kinase
DOX: doxorubicin
EGR1: early growth response-1
EMT: epithelial-mesenchymal transformation
ER: estrogen receptor
ERCC: excision repair cross-complementing gene
ERS: endoplasmic reticulum stress
FASN: fatty acid synthase
FBP1: fructose 1, 6-Bisphosphatase 1
FGFR: fibroblast growth factor receptor
GBC: gallbladder carcinoma
GBM: glioblastoma
GSCs: glioma stem cells
HNSCC: head and neck squamous cell carcinoma
HCC: hepatocellular carcinoma
HPV: human papillomavirus
IR: ionizing radiation
JAK2: Janus-activated kinase 2
Mcl-1: myeloid cell Leukemia 1
MDR-1: multidrug resistance gene-1
MM: multiple myeloma
MMOP: mitochondrial transmembrane potential
MMP: matrix metalloproteinases
MRSA: methicillin-resistant *Staphylococcus aureus*
MSCs: mesenchymal stem cells
MTH1: MutT homologue 1
NAC: N-acetylcysteine
NCOR2: nuclear receptor corepressor 2
NER: nucleotide excision repair
NHL: non-Hodgkin's Lymphoma
NO: nitric oxide
NUDT1: nudix hydrolase 1
P2X7R: permeable channel P2X7 receptor
PC: pancreatic cancer
Ph: philadelphia
ROS: reactive oxygen species
TNBC: triple-negative breast cancer
UHRF1: ubiquitin-like protein containing PHD and RING domains 1
VM: vasculogenic Mimicry
STAT3: signal transducer and activator of transcription 3
